# Connecting the dots between different networks: miRNAs associated with bladder cancer risk and progression

**DOI:** 10.1186/s13046-019-1406-6

**Published:** 2019-10-29

**Authors:** Cornelia Braicu, Rares Buiga, Roxana Cojocneanu, Mihail Buse, Lajos Raduly, Laura Ancuta Pop, Sergiu Chira, Liviuta Budisan, Ancuta Jurj, Cristina Ciocan, Lorand Magdo, Alexandru Irimie, Florentin Dobrota, Bogdan Petrut, Ioana Berindan-Neagoe

**Affiliations:** 1 Research Center for Functional Genomics Biomedicine and Translational Medicine, “Iuliu Hatieganu” University of Medicine and Pharmacy, Cluj-Napoca, Romania; 2 Department of Pathology, “Prof. Dr. Ion Chiricuta” Oncology Institute, Cluj-Napoca, Romania; 3 Department of Pathology, “Iuliu Hatieganu” University of Medicine and Pharmacy, Cluj-Napoca, Romania; 4 MedFuture Research Center for Advanced Medicine, “Iuliu Hatieganu” University of Medicine and Pharmacy, Cluj-Napoca, Romania; 5 Department of Surgery, “Prof. Dr. Ion Chiricuta” Oncology Institute, Cluj-Napoca, Romania; 6 Department of Surgical Oncology and Gynecological Oncology, “Iuliu Hatieganu” University of Medicine and Pharmacy, Cluj-Napoca, Romania; 7 Department of Urology, “Iuliu Hatieganu” University of Medicine and Pharmacy, 400012 Cluj-Napoca, Romania; 8 Department of Urology, “Prof. Dr. Ion Chiricuta” Oncology Institute, Cluj-Napoca, Romania; 9 Department of Urology, “Iuliu Hatieganu” University of Medicine and Pharmacy, 400012 Cluj-Napoca, Romania; 10 Department of Functional Genomics and Experimental Pathology, “Prof. Dr. Ion Chiricuta” Oncology Institute, Cluj-Napoca, Romania

**Keywords:** Bladder cancer, miRNA, Mutation, miRNA-mRNA network

## Abstract

**Background:**

Bladder cancer (BC) is a common urothelial malignancy, characterized by a high recurrence rate. The biology of bladder cancer is complex and needs to be deciphered. The latest evidence reveals the critical role of the non-coding RNAs, particularly microRNAs (miRNAs), as vital regulatory elements in cancer.

**Method:**

We performed a miRNAs microarray using paired tissues (tumor and adjacent normal bladder tissue), followed by the validation with qRT-PCR of five selected transcripts. Additional next-generation sequencing investigation established the interconnection among the altered miRNAs and mutated genes. Based on the overlapping between TCGA data and data obtained in the study, we focused on the systematic identification of altered miRNAs and genes mutated involved in bladder cancer tumorigenesis and progression.

**Results:**

By overlapping the miRNAs expression data, the two patient cohorts, we identified 18 miRNAs downregulated and, 187 miRNAs upregulated. qRT-PCR validation was completed using a selected panel of two downregulated (miR-139-5p and miR-143-5p) and three up-regulated miRNAs (miR-141b, miR-200 s or miR-205). Altered miRNAs patterns are interrelated to bladder tumorigenesis, allowing them to be used for the development of novel diagnostic and prognostic biomarkers. Three EMT-related upregulated miRNAs have an essential role in the molecular mechanisms, specifically key processes underlying tumorigenesis, invasion and metastasis. Using the Ampliseq Cancer Panel kit and Ion Torrent PGM Next-Generation Sequencing an increased mutation rate for *TP53, FGFR3, KDR, PIK3CA* and *ATM* were observed, but the mutational status for only TP53 was correlated to the survival rate. The miRNAs pattern, along with the gene mutation pattern attained, can assist for better patient diagnosis.

**Conclusion:**

This study thereby incorporates miRNAs as critical players in bladder cancer prognosis, where their altered gene expression profiles have a critical biological function in relationship with tumor molecular phenotype. The miRNA-mRNA regulatory networks identified in BC are ripe for exploitation as biomarkers or targeted therapeutic strategies.

## Background

Bladder cancer (BC) represents a global problem for the urinary tract, statistically situated in fifth place in terms of mortality and morbidity [1]. BC incidence is higher in aged populations and is correlated with toxic environmental agents, in particular with smoking [2]. The prognosis of BC is unfavorable, with a high percentage of disease recurrence regardless of treatment; these treatments include surgery, chemotherapy or, a combination of these two [1]. About 70–75% of BC cases are defined as non-muscle invasive tumors, subclassified into low or high-grade tumors [3–5]. The identification of high-risk tumors is imperative and requires intravesical therapy as a standard practice [6].

BC is a multifactorial disease where both exogenous and endogenous factors are eessential in early carcinogenesis, disease progression but also responsible for the high recurrence rate, correlating to a specific mutation pattern [7]. Therefore, new biomarkers for early diagnosis of bladder cancer, prognostic markers for its recurrence and predictive markers for response/overall survival are needed with great urgency [8]. Molecular markers can provide vital information to refine the optimal treatment, which in turn permits a good patient prognosis [5].

MicroRNAs (miRNAs) are a novel class of short non-coding RNA sequences that have around 19–25 nucleotide length [8, 9]. These short non-coding structures can regulate gene expression and interfere with vital cellular pathways without being translated into proteins, including in cancer biology. A miRNA can target multiple genes, several miRNAs can target a gene and this gives rise to complex interaction networks; presently, the exact interactions have yet to be determined especially in relationship to the mutational status [8, 10]. Accordingly, miRNAs are essential candidates for both diagnosis and prognosis due to their oncogenic or tumor suppressor functions. Hence, miRNAs profiling studies from different tissues represent an excellent alternative application for these short sequences as biomarkers with clinical significance [11]. The main advantage is the high stability of these transcripts, remaining unaltered during the surgical resection procedure (TURB), which is often associated with a high degradation rate [12, 13]. Another advantage is the full range of tools and methods for miRNAs profiling (microarray, next-generation sequencing or Nanostring) or validation (qRT-PCR or in situ hybridization) [8].

Previous studies have examined miRNAs in BC; however, very few cases examine the global miRNA expression patterns by microarray in paired samples with subsequent overlapping using TCGA miRNA data. We utilized this combined data set to identify specific pathways associated with BC. Following the profiling of bladder cancer samples, we used Ion Torrent Next Generation Sequencing Cancer panel to determine the most relevant mutations in our patient cohort and TCGA dataset. The correlation of this data permits a better understanding of the interconnected regulatory networks that appear to have significant biological meaning in terms of tumor molecular phenotype and gene expression profiles; all this to be exploited as candidates for future therapies or as prognostic/diagnostic biomarkers.

## Material and methods

### Sample collection

Between 2014 and 2016, we collected the tumoral BC tissue along with the adjacent healthy tissue from patients, only after obtaining informed consent from each patient and approval by the institutional ethics committee of the Iuliu Hatieganu University of Medicine and Pharmacy, Cluj-Napoca, Romania (UMPh), with the authorization no. 673A/20.11.2012. We stored transurethral resections of bladder tumor (TURBT) tissues in liquid nitrogen until sample processing and RNA extraction. When surgical and pathological procedures permitted, surgeons collected the healthy tissue, adjacent from the tumor, from each patient. We initially evaluated the expression of Her2 and TP53 using the standard immunohistochemistry staining protocol. The paired (healthy and tumor) tissue samples later used for the microarray and next-generation sequencing analyses are referred to as UMPh patient cohort. The second group of samples collected was for the qRT-PCR validation and named the validation set; we collected this additional patient cohort.

### Sample processing and microarray evaluation

The total RNA extraction and isolation from 23 paired samples (normal and tumoral bladder tissue) is done using the TriReagent (Sigma-Aldrich) protocol. NanoDrop-1000 spectrophotometer was used to measure the concentration of RNA. The microarray probes were synthesized from equal quantities of 100 ng of total RNA, by using miRNA microarray protocol based on version 3.1 of September 2015 (Agilent Technologies) which included complete labeling and hybridization kit (cat no. 5190–0456 Agilent) and a purification step with Micro Bio-Spin P-6 Gel Column (Biorad). The SureScan Microarray Scanner (Agilent Technologies) scanned the microarray slides and Feature Extraction 12.0 software performed data extraction. The last step in the microarray evaluation was to identify the primary altered miRNAs. Gene Spring GX v.13.0, applying a fold change (FC) threshold of 2 moderated t-test and False Discovery Rate correction (*p*-valued ≤0.05), analyzed the microarray data and generated the comparisons of low-grade versus high-grade tumor tissues; low-grade tumor versus healthy tissues; high-grade tumors versus healthy tissues (data available on Arrayexpress, ID: E-MTAB-8356).

### Bladder cancer TCGA data analysis

We performed a supplementary analysis at the third level of miRNAs-sequencing from 409 bladder tumors and 19 healthy tissues, adjacent to the tumors, obtained from the TCGA data portal (https://tcga-data.nci.nih.gov/tcga/). The data were analyzed in GeneSpring GX v.13.0, applying the previous defined cut-off value.

### miRNA qRT-PCR evaluation on tissue samples

We selected to validate three upregulated transcripts (miR-23a, miR-141-3p and miR-205-5p) and two downregulated transcripts (miR-139-5p, and miR-143-5p) from the paired tissue samples. RNA was extracted using TriReagent based method for qRT-PCR validation was performed on 18 healthy bladder tissues and 18 bladder tumor tissues. We performed the cDNA synthesis using a 7.5 μl of reverse transcription mixture containing 0.72 μl of RT primer, 50 ng of total RNA and 0.5 μl of MultiScribe Reverse Transcriptase, 0.75 μl Reverse Transcription Buffer (10×), 0.075 μl dNTPs (100 mM), 0.1 μl of RNase Inhibitor according to Taqman MicroRNA Reverse Transcription Kit (Applied Biosystems) protocol. The cDNA mixture is incubated in PCR tubes for 30 min at 16 °C, 30 min at 42 °C and 5 min at 85 °C. qRT-PCR was performed using the in ViiA7 (Applied Biosystems) PCR machine with a total volume of reaction mix of 12.5 μl; this reaction mix consists of 6.25 μl of cDNA (diluted 1:6 with nuclease-free water), 5.63 μl of SSoAdvanced Universal Probe Supermix (Bio-Rad) and 0.73 μl primers for each miRNA. The reactions were set up as follows: initial denaturation step at 95 °C for 180 s, followed by 39 cycles of 95 °C for 5 s and, lastly, 60 °C for the 30s. The expression level of each miRNA is calculated by the threshold cycle (C_T_). The relative expression level was calculated using –ΔΔC_T_ method and U6 for normalization.

### TP53 evaluation by qRT-PCR on bladder tissue samples

Lab technicians synthesized the cDNA using High-Capacity cDNA Reverse Transcription Kit (Applied Biosystems). The reaction preparation of qRT-qPCR used the SYBR Select Master Mix (Life Technologies) and executed using ViiA7. The following conditions were used: 95 °C for 2 min, 40 cycles of 95 °C for 10 s and 60 °C for 1 min. The FC of gene expression was calculated with the ΔΔC_T_ method, using B2M as the housekeeping gene.

### Next-generation sequencing of bladder cancer samples

A number of 22 bladder cancer samples, the same ones analyzed by microarray (except one samples from microarray patien cohort with low DNA concentration), were sequenced using Ion Ampliseq Cancer Panel and Ion Torrent PGM Next Generation Sequencing (Thermo Fischer Scientific); this panel contains the most relevant hot spot mutation. The amplicon libraries were prepared with 20 ng of DNA and the Ion Ampliseq™ Library Kit 2.0 (Life Technologies) and this was followed by a purification step using AMpure XP Beads (Beckman Coulter). Lastly, Qubit 2.0 was used for the quantification using Qubit HS DNA kit. For sequencing, four bar-coded 100pM-diluted libraries were used for each Ion 316 Chip (Thermo Fischer Scientific). Ion Torrent PGM Machine (Thermo Fischer Scientific) performed the sequencing, using the Ion PGM HI-Q Sequencing 200 kit. The software Torrent Suit 5.6 and Ion Reporter 5.6 executed the bioinformatics analysis, specifically for data trimming alignment and variant calling.

### Functional analysis and target genes identification

The IPA analysis determined the miRNAs with altered expression levels to identify the most relevant networks, altered pathways and their respective biological significance. For the identification of the target genes most relevant to our miRNAs, the following database and webservers were used: miRTarBase (https://bio.tools/mirtarbase); miRNet (https://www.mirnet.ca/); and miRtargetLink (https://ccb-web.cs.uni-saarland.de/mirtargetlink/).

## Results

### Bladder cancer patients’ statistics

**Fig. 1:**
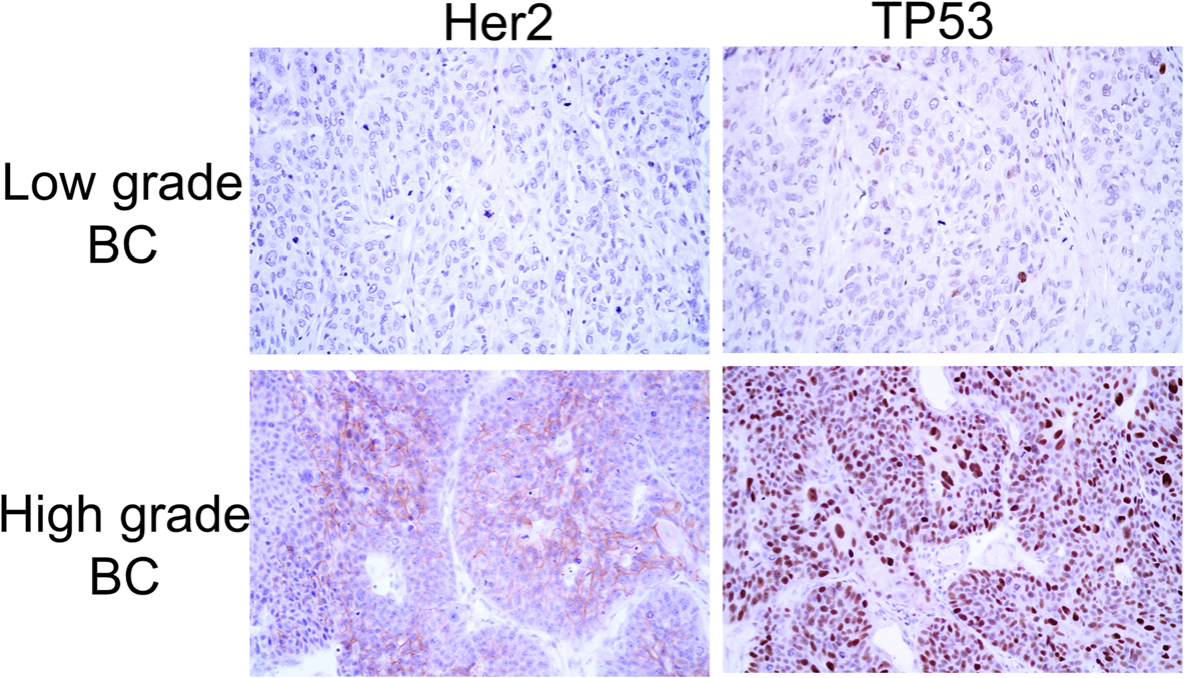
Expression of *Her2* and *TP53* in low grade and high bladder tumor samples using the standard immunohistochemistry staining protocol (400x magnification)

We determined the initial miRNA profiling using the WHO-2016 classification and the most frequent histological type, urothelial carcinoma. More specifically for the microarray study, the patients’ samples were graded as low or high and, additionally, through immunohistochemistry (IHC), the expression level of *Her2* and *TP53* was determined. Figure 1 exemplifies a low grade negative stained case for *TP53* and *Her2* compared to a high grade positive stained case for TP53 and Her2.

Table 1 presents the demographics and patient characteristic selected for the microarray. Of the 23 patients included in the microarray cohort, the average age is 64.04 ± 11.72, consisting of 5 females with an age average of 56.80 ± 6.30 and 18 males with an age average of 66.05 ± 12.20. The average age for the qRT-PCR cohort of 18 patients (5 females and 13 males), is respectively 66 ± 9,59 (57,2 ± 7,98 for females, respectively 69,38 ± 8.03 for males), which can be seen in Table 2. We classified the patient cohort selected for the profiling and qRT-PCR validations into two main subtypes: low-grade non-invasive tumors and high grade advanced tumors.

**Table 1:** Demographic and histopathological characteristics of the 23 participants used for miRNA microarray evaluation

No.	Sex	Age	Histopathological diagnosis	Histopatological stage
1	M	81	high grade urothelial carcinoma	pT2
2	M	71	low grade urothelial carcinoma	pTa
3	M	61	high grade urothelial carcinoma	pT1
4	F	53	low grade urothelial carcinoma	PTa
5	F	50	low grade urothelial carcinoma	pTa
6	M	78	low grade urothelial carcinoma	pTa
7	F	54	high grade urothelial carcinoma	pT2
8	M	45	high grade urothelial carcinoma	pT4
9	M	67	low grade urothelial carcinoma	pT1
10	F	63	high grade urothelial carcinoma	pT2
11	M	43	low grade urothelial carcinoma	pTa
12	M	60	low grade urothelial carcinoma	pTa
13	M	75	low grade urothelial carcinoma	pTa
14	M	75	low grade urothelial carcinoma	PTis
15	M	61	high grade urothelial carcinoma	pT4
16	M	93	high grade urothelial carcinoma	pT3a
17	M	65	high grade urothelial carcinoma	pT2
18	M	58	high grade urothelial carcinoma	pT2
19	M	57	high grade urothelial carcinoma	pTa
20	F	64	low grade urothelial carcinoma	pTa
21	M	61	low grade urothelial carcinoma	pT1
22	M	70	high grade urothelial carcinoma	pT2
23	M	68	high grade urothelial carcinoma	pT2

**Table 2:** Demographic and histopathological characteristics of the eighteen participants used for qRT-PCR data validation

Patient code	Sex	Age	Histopathological diagnosis	Histopatological stage
1	M	82	high grade urothelial carcinoma	pT2
2	F	68	high grade urothelial carcinoma	pT1
3	M	59	low grade urothelial carcinoma	pT1
4	M	77	high grade urothelial carcinoma	pT1
5	M	65	high grade urothelial carcinoma	pTa
6	F	54	high grade urothelial carcinoma	pT4a
7	M	58	high grade urothelial carcinoma	pT2
8	M	75	high grade urothelial carcinoma	pT2
9	M	67	low grade urothelial carcinoma	pTa
10	F	52	high grade urothelial carcinoma	pTa
11	M	73	high grade urothelial carcinoma	pT1
12	F	63	high grade urothelial carcinoma	pT2
13	M	67	high grade urothelial carcinoma	pT2
14	M	58	high grade urothelial carcinoma	pTa
15	M	68	high grade urothelial carcinoma	pT1a
16	M	74	high grade urothelial carcinoma	pT1
17	M	79	high grade urothelial carcinoma	pT3b
18	F	49	high grade urothelial carcinoma	pTa

### Evaluation of tissue miRNA patterns in bladder cancer patients

**Fig. 2:**
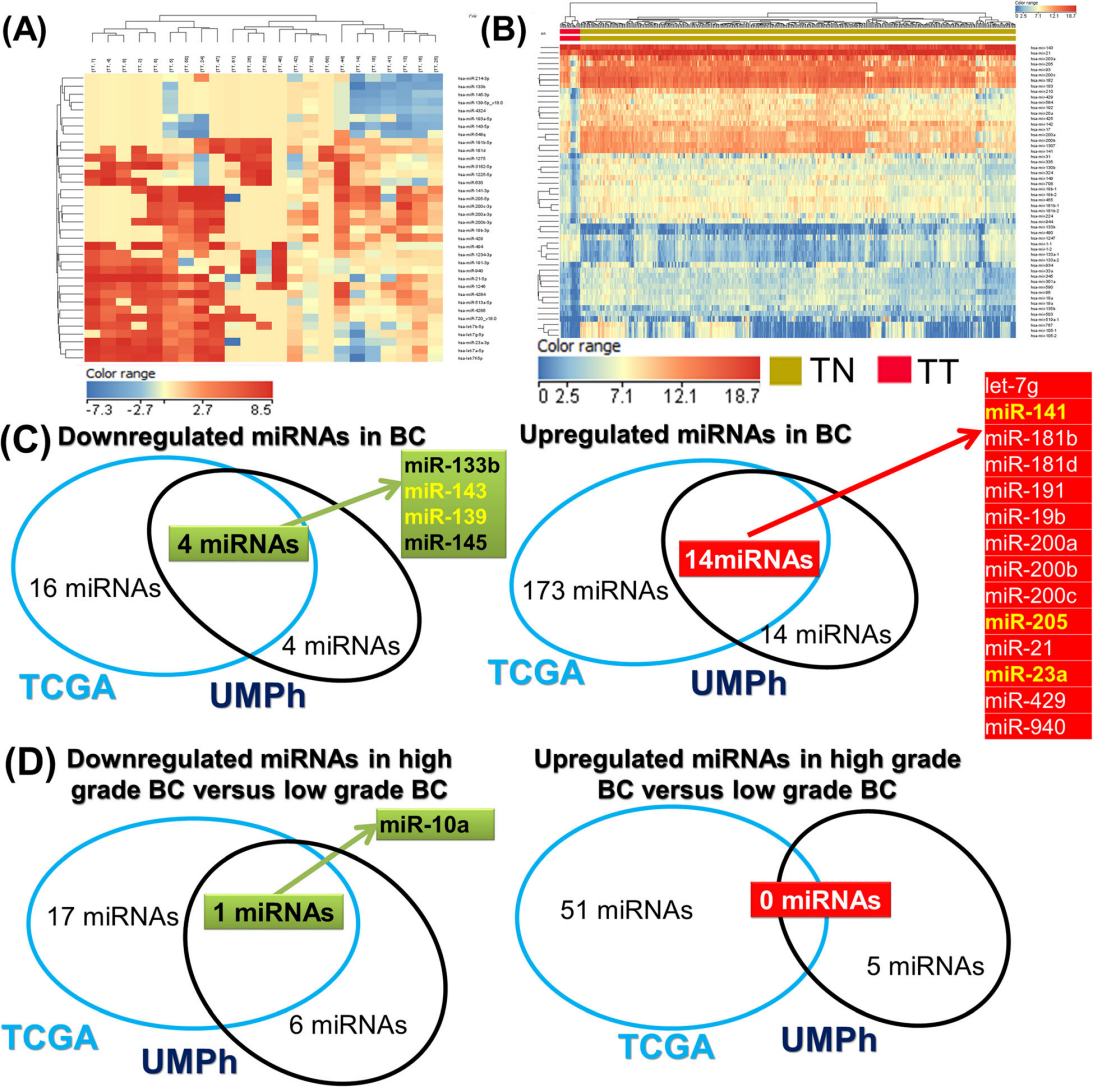
The tissue-specific signatures of miRNAs in bladder cancer. **a** miRNA Heatmap emphasizing the altered miRNA signatures in 409 tumor tissue versus 19 healthy tissue taken from the TCGA bladder cancer patient cohort. **b** miRNA Heatmap highlighting the modified miRNA pattern when comparing tumor versus healthy tissues, representing the 23 paired bladder tissues from the UMPh patients’ cohort. **c** Venn diagram of the statistically determined (FC ± 2 and *p*-value ≤0.05) upregulated and downregulated miRNA expressions by overlapping the UMPh and TCGA patient cohorts; **d** Venn diagram of the UMPh and TCGA patient cohorts from the statistically determined (FC ± 2 and *p*-value≤0.05) upregulated and downregulated miRNA expressions exhibited in high grade tumor versus healthy tissues

The present profiling investigation uses Agilent 60-mer SurePrint microarray technology that contains capture probes to characterize the expression of 2549 mature human miRNAs, obtained from the annotated miRbase 16. This profiling investigation leads to the identification of the most relevant altered miRNAs based on an analysis of paired samples: bladder cancer tumor tissues (TT) versus adjacent healthy tissues (TN). It identified 8 down-regulated and 28 up-regulated miRNAs in the UMPh patient cohort (Additional file 1: Table S1). The Heatmap analysis presenting the altered miRNAs in tumor tissue for UMPh patient cohort is showed in Fig. 2a; the blue color denoting downregulation while the red color signifies overexpression.

To strengthen and improve the accuracy of the miRNAs profiling investigation, we performed an additional TCGA analysis of 409 tumors and 19 tumor-adjacent healthy tissues (Additional file 2: Table S2). The cut-off value was FC ± 2 and *p*-value≤0,05 for this analysis, from which 18 downregulated and 187 upregulated miRNAs were identified (Additional file 3: Table S3). The Heatmap for this data is shown in Fig. 2b. We overlapped the miRNAs profiling data obtained from the two datasets mentioned above. The Venn diagram represented in Fig. 2c shows 14 upregulated and 4 downregulated miRNAs. For the analysis of the high versus low-grade bladder cancers (UMPh: Additional file 4: Table S4; TCGA: Additional file 5: Table S5), only one commonality, the downregulation of miR-10a, was identified (Fig. 2d).

**Fig. 3:**
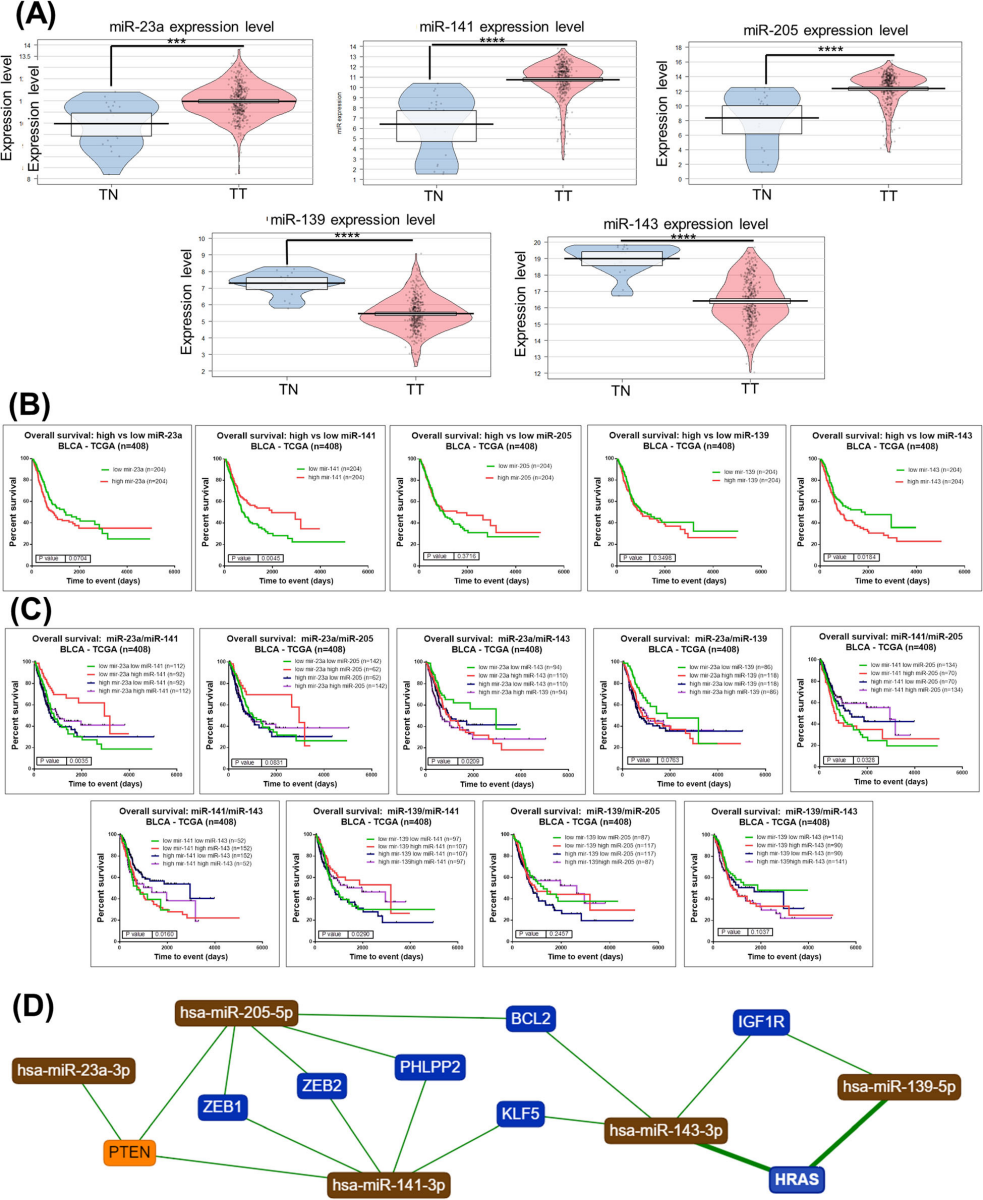
The respective miRNA expression level determined from the TCGA data on bladder cancer. **a** Expression levels presented as pirate plots based on TCGA data for five selected altered miRNA transcripts. **b** Kaplan–Meier survival curve for each selected miRNA transcript altered in bladder cancer based on TCGA data; **c** Kaplan–Meier survival curve for combinations of two miRNA transcripts from the five selected (*p ≤ 0.05, **p ≤ 0.01, ***P ≤ 0.001, Ns: not statistically significant, *p* > 0.05). **d** The network interconnection among the selected miRNA transcripts and genes generated using miRtargetLink (https://ccb-web.cs.uni-saarland.de/mirtargetlink/)

Given the context from the information displayed in Fig. 3, five miRNAs were selected for further validation based on the statistically significant FC obtained from our paired bladder tissues samples and the following criteria: the two most downregulated miRNAs (miR-143 and miR-139); the highly upregulated miRNAs related to EMT that have been less studied (miR-141 and miR-205); and one intermediary upregulated miRNA (miR-23a).

Figure 3a shows the Pirate plots that were generated using the TCGA data for the five selected miRNAs (overexpressed transcripts: miR-23a, miR-141 and miR-205; downregulated transcripts: miR-139 and miR-143). The related survival curves for each miRNA transcript can be seen Fig. 3b while Fig. 3c displays the survival curves for combinations of two miRNAs. We observed an overall superior survival rate for the following three cases: high expression of miR-141 (*p* = 0.0045); low expression of miR-143 (*p* = 0.0184); and low expression of miR-143 combined with high expression of miR-141 (*p* = 0.0163). These altered miRNAs are directly interconectd via key genes regulated apoptosis or invasion related genes as can be emphases by network generated using miRtargetlink (Fig. 3d).

### qRT-PCR data validation

**Fig. 4:**
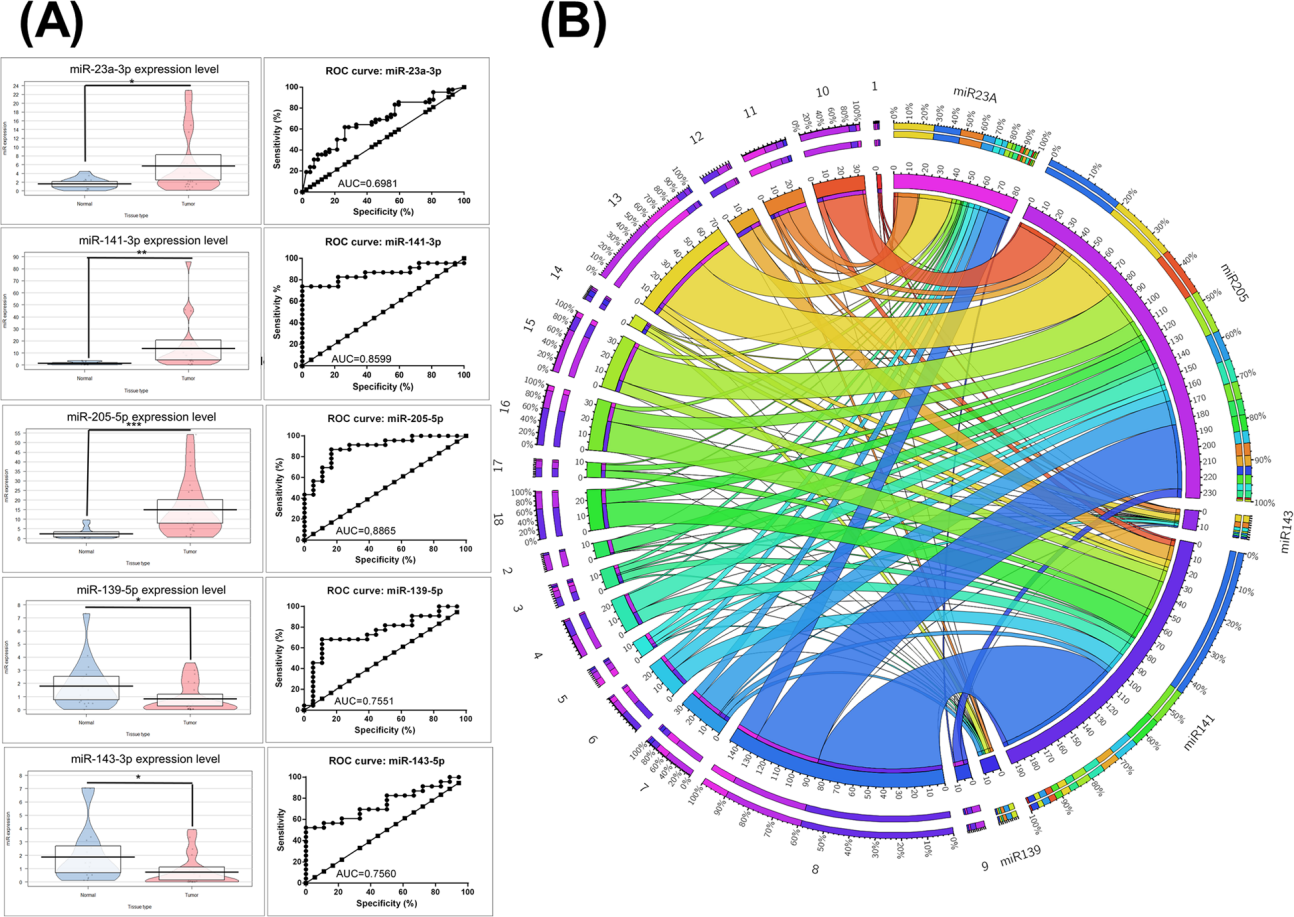
qRT-PCR data validation. **a** Left panel of Pirate Plots presents the expression level for miR-23a-3p, miR-139-5p, miR-141-3p, miR-143-5p and miR-205-5p in the new validation set comprised of healthy (*n* = 18) and tumor tissues (*n* = 18). The data was normalized using U6 based on ΔΔC_t_ method (*p < 0.05, **p < 0.01, ***p < 0.001). Right panel of graphs presents ROC curves for each selected miRNA’s specificity and sensitivity (ROC: receiver-operating characteristic, AUC: area under ROC curve). **b** Circos diagram representing the expression level for the validated miRNA transcripts in tumoral tissues of the new patient cohort

qRT-PCR is one of the most relevant and standardized approaches to validate microarray data. The new patient cohort used for the qRT-PCR consisted of 18 healthy tissues and 18 tumor tissues. From the common list of miRNAs between UMPh patients’ cohort and TCGA data, we selected two downregulated (miR-139-5p and miR-143-5p) and three up-regulated (miR-23a-3p, miR-141-3p, and miR-205-5p) to validate as independent prognostic markers. For miRNAs normalization, the ΔΔCt method with U6 as reference/normalizer; qRT-PCR validation data of the new additional patient cohort is presented in Fig. 4a. A ROC curve was constructed to estimate the sensitivity and specificity of these transcripts as biomarkers for bladder cancer, the highest AUC value being for miR-141 (0.8599) and miR-205 (0.8865). Circos diagram can be seen in Fig. 4b and the following observations were made: miR-205-5p and miR-141-3p are very heterogeneous providing expression level value, that should be considered as possible biomarker. miR-143-5p and miR-139-5p are very homogenous offering a potential advantage as biomarkers; and as expected with our relatively intermediary-expressed upregulated microRNA, miR-23a lies between the previously mentioned miRNAs.

### Mutation signatures are characteristic of bladder cancer tissues

**Fig. 5:**
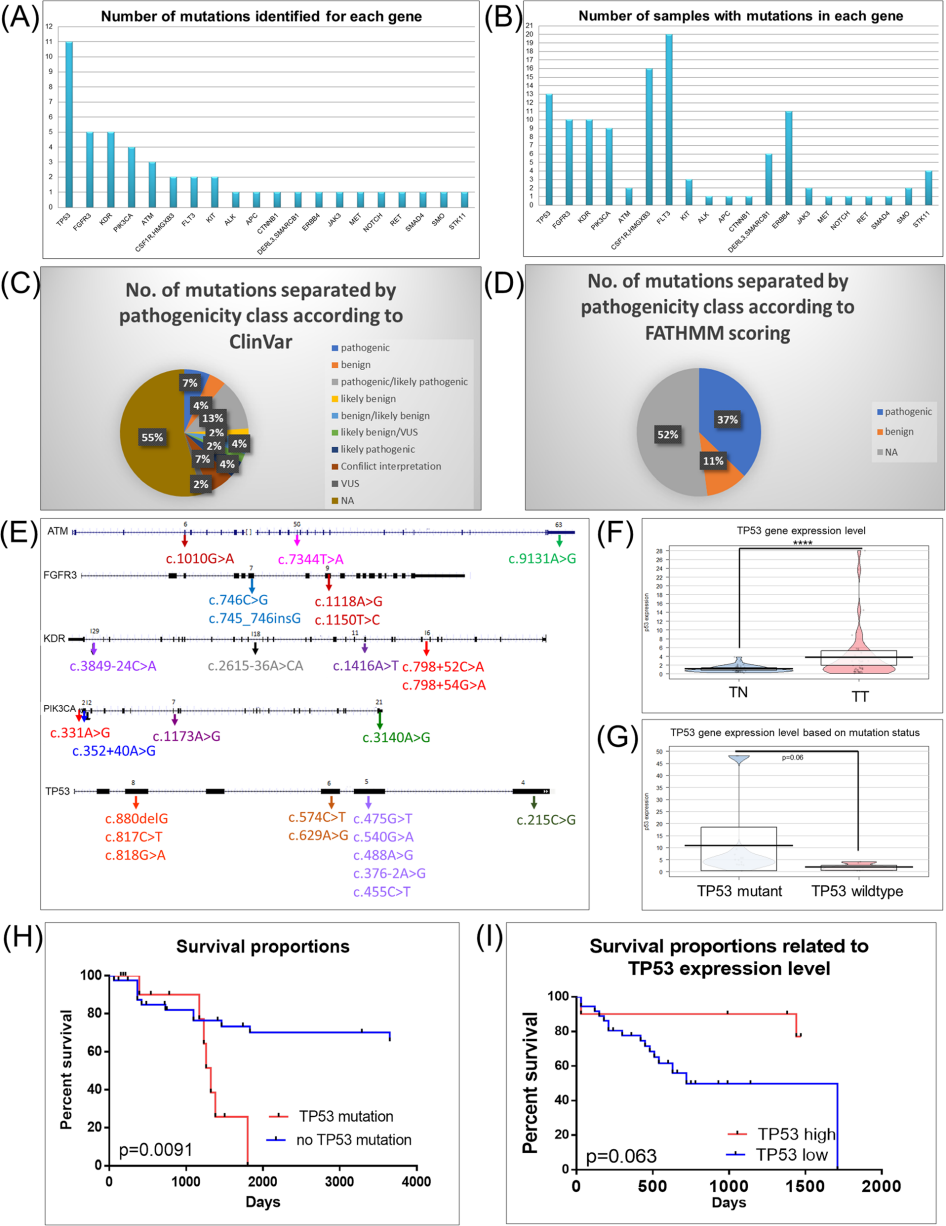
Mutation patterns in bladder cancer patients evaluated using Ion Ampliseq Cancer panel kit. **a** Bar Graph of the number of mutations identified for each gene in the Ion Ampliseq Cancer kit; **b** Bar Graph of the mutation frequency of each gene in the Ion Torrent Cancer panel from the analyzed UMPh patient samples; **c** Pie graph of the identified mutations separated by pathogenicity according to ClinVar classification; **d** Pie graph of the identified mutations separated by pathogenicity according to FATHMM scoring and classification; **e** Localization of mutations in each of the gene sequences for the most frequent mutated genes in bladder cancer patients; **f** qRT-PCR evaluation of TP53 gene expression level in all healthy tissues (*n* = 41) and bladder tumor tissues (*n* = 41); this cohort means the 23 samples from the UMPh patient cohort and 18 samples from the validation set. **g** Pirate Plot of the *TP53* expression level in wild-type and *TP53*-mutated samples; **h** Kaplan-Meier curve of overall survival rate based on mutation status for TP53 mutated gene from the UMPh patient cohort; **i** Kaplan-Meier curve of overall survival rate based on TP53 expression levels from the UMPh patient cohort

We analyzed the mutation patterns for tumor tissues from the UMPh cohort of 22 patients diagnosed with bladder cancer (Additional file 9: Table S6). An increased mutation rate is shown for *TP53, FGFR3, KDR, PIK3CA* and *ATM*. Figure 5a presents the number of mutations identified in each gene; meanwhile, Fig. 5b shows the mutations frequency for each gene in the analyzed samples. Using NCBI ClinVar or FATHMM, the mutations exhibited were classified based on pathogenicity, meaning as pathogenic, benign/non-pathogenic or not applicable/unknown (NA) (Fig. 5c). A significant percentage of the mutations were classified with unknown (NA) role (55%) and 7% are pathogenic based on the ClinVar classification. In comparison, using the FATHMM scoring 52% were classified as unknown (NA) while 37% of the mutations were classified as pathogenic (Fig. 5d). The mutated genes in bladder cancer have both intronic and exonic localizations. The mutation localizations for the most relevant genes (*ATM, FGFR3, KDR, TP53* and *PIK3CA*) are presented in Fig. 5e. For the case of *TP53*, an increased survival rate for patients expressing *TP53* wild-type versus those expressing mutant was observed; for the rest of the frequently mutated genes there was no statistically significant data. The frequency of mutations in our pattern exhibited is consistent with the frequent mutations in 200 bladder cancer cases from the TCGA; OncoGrid summarizes the 200 most mutated bladder cancer cases and top 50 mutated genes. From the TGCA data and the frequent mutated genes obtained, the overall survival rate for the case of patients expressing mutated and wild-type genes can be seen Additional file 6: Figure S1. No statistically significant correlation between the survival rate and mutational status was observed for *AKT, ATM, CTNNB1, FGFR3, TP53, KIT, MET, PIK3CA* and *SMO*.

### Evaluation of TP53 expression using qRT-PCR

The calculation of gene expression FC used the ΔΔC_T_ method and *B2M* as the housekeeping gene (Fig. 5f). Figure 5f presents relative expression level of TP53 when comparing bladder tumor and normal tissues. There was no correlation among the expression level of TP53and their status (Fig. 5g). An additional graph of TP53 expression level using a median cut-off (50% above or low) to separate the expression levels into high or low and TP53 mutational status can be seen in Fig. 5e or based on the expression level (Fig. 5i). We observed an increase in survival rate for the case of TP53 wild type patients versus mutant TP53 (*p* = 0.0091).

### Identification of miRNAs functional pathways related to bladder cancer tumorigenesis

**Fig. 6:**
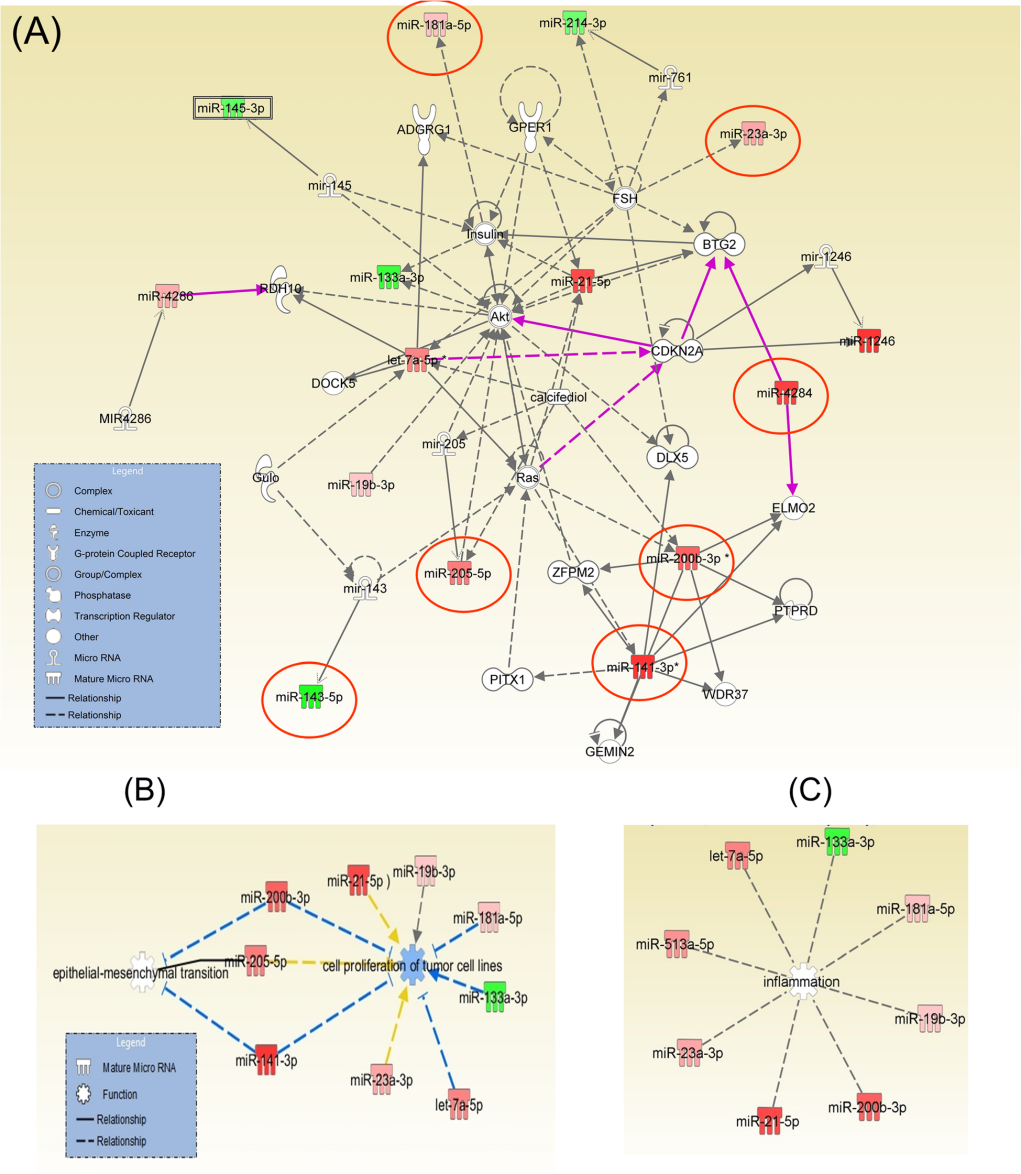
The bladder cancer specific miRNA-gene regulatory network of the genes found by NGS and their miRNA target. **a** Overlapping of most relevant miRNA molecular networks involved in bladder cancer. The overexpressed miRNAs are highlighted in red, the downregulated miRNAs are marked in green, and the most relevant target genes determined from the literature are in grey. A solid connecting line represents a direct action while a dotted line signifies an indirect action. **b** IPA analysis for the identification of miRNAs related to epithelial to mesenchymal transition (EMT) (**c**) IPA analysis for identification of miRNAs related to inflammation

The miRNAs with an altered expression level were input into the specialized software IPA (Ingenuity Pathways Analysis). This analysis highlights the main altered networks indicating disease or biological function based on the miRNA signatures from the bladder cancer patients and the interconnection with target genes. Therefore, 9 relevant molecular networks were identified for bladder cancer as can be seen in Table 3. Furthermore, Table 4 shows the statistically significant biological, cellular and molecular functions as well as diseases that these altered miRNAs are associated to. This statistical correlation is based on the number of molecules altered within a given network. The data from Table 4 was used to generate graphical representation of molecular networks using IPA, which displays the nodal genes for the altered miRNAs in bladder cancer. This improves identification of miRNA-gene interactions and determines their nature as direct or indirect. Figure 6a presents an overlap of the most important networks in terms of the connection between the altered miRNAs and target genes. Altered miRNAs target genes critical to regulating tumour progression and cell fate are particularly important for patient prognosis. Once again, this highlights the connection of the uncovered genes *TP53, AKT, RAS, CDKN2A* or *CDK2* to cell fate, as important regulators. These genes are interconnected with the validated miRNAs which are highlighted with red circles. Another important mechanism in carcinogenesis is the transition from epithelial to mesenchymal (EMT) phenotype (Fig. 6b). It was determined that *AKT *is directly related to *ZEB2* that interacts with miR-205-5p, miR-141-3p and miR-200b-3p. These miRNAs have highest expression levels, extracted from the top altered transcripts of both patient cohorts (found in the TCGA as well as our experimental data). Thus, genes were identified that are involved in carcinogenesis or responsible to apoptotic resistance. These genes are recognized to have prognostic role in colorectal cancer (*RAS*) or related to the progression of bladder cancer (*AKT*).* RAS* and *AKT* have been shown to be related to miR-21. Figure 6c highlights the relevant transcripts related to inflammation, based on the literature information including the IPA.

**Table 3:** The altered networks based on the miRNA signature of the bladder cancer participants, where the relevance score was generated using IPA. Each network integrates miRNAs along with the most relevant target genes, indicating disease or biological function, based on which the score is calculated

ID	Top Diseases and Functions	Score	Focus Molecules	Molecules in Network
1	Organismal Injury and Abnormalities, Reproductive System Disease, Cancer	29	12	↑let-7a-5p*, mir-143, mir-145, mir-205, mir-761, ↓miR-133a-3p, ↑miR-141-3p*, ↓miR-143-5p, ↓miR-145-3p, ↑miR-181a-5p, ↑miR-19b-3p, ↑miR-200b-3p*,↑ miR-205-5p, ↑miR-21-5p, ↓miR-214-3p, ↑miR-23a-3p, PITX1, PTPRD, Ras, RDH10, resolvin D1, WDR37, ZFPM2
2	Cancer, Organismal Injury Abnormalities, Cell Cycle	6	3	BRF1, CDK2, Ck2, MIR3162, mir-25, mir-193, mir-506, ↓ miR-193a-5p, ↑miR-3162-5p, ↑miR-513a-5p, RNA polymerase iii, SSB, TP53
3	Organismal Injury and Abnormalities, Reproductive System Disease, Developmental Disorder	3	1	mir-548, ↓miR-548q
4	Organismal Injury and Abnormalities, Reproductive System Disease, Cancer	3	1	mir-191, ↑miR-191-3p
5	Developmental Disorder, Hereditary Disorder, Organismal Injury, Abnormalities	3	1	miR-1225, ↑mir-1225-5p
6	Cancer, Cell Cycle, Cell Death and Survival	3	1	CDKN2A, mir-1246, ↑miR-1246
7	Cancer, Gastrointestinal Disease, Organismal Injury and Abnormalities	3	1	MIR1234, MIR7170, ↑miR-1234-3P
8	Cancer, Gastrointestinal Disease, Organismal Injury and Abnormalities	3	1	mir-630, ↑miR-630, TMEM8B
9	Cancer, Connective Tissue Disorders, Hematological Disease	2	1	miR-4665, miR-6941, miR-7023, miR-7024, miR-7119, miR-1275, ↑miR-1275, YBX1

**Table 4:** List of diseases and functions at the biological, molecular or cellular level that are the most representative of the altered molecules exhibited in bladder cancer. The selected IPA score based on *p*-value and number of molecules altered in bladder cancer; more specifically, *p*-values is calculated based on the number of molecules altered in a given pathway divided by the total number of molecules from a specific pathway or biological processes

Function	Name	*p*-value	#Molecules
Top Diseases and Bio Functions	Organismal Injury and Abnormalities	4.85E-02 – 5.80E-14	16
	Reproductive System Disease	3.30E-02 – 5.80E-14	12
	Cancer	4.62E-02 – 1.75E-13	14
	Inflammatory Disease	1.49E-02 – 2.42E-08	9
	Inflammatory Response	4.64E-03 – 2.42E-08	8
Molecular and Cellular Functions	Cellular Development	4.99E-02 – 1.60E-05	9
	Cellular Growth and Proliferation	4.99E-02 – 1.60E-05	9
	Cellular Movement	2.15E-02 – 4.20E-05	5
	Cell Death and Survival	4.99E-02 – 1.55E-03	7
	Cell Morphology	2.15E-02 – 1.55E-03	3

### miRNA target genes involved in bladder cancer

In order to identify the biological significance of the altered miRNA expression for bladder cancer, miRTarBase was used to identify validated target genes. The target genes relating to the most upregulated miRNAs were grouped into specific families. These miRNA families have an important role in the regulation of translational processes and, through their identification, relevant hallmarks of cancer can be correlated. Using the miRnet database, we determined the initial miRNA-target interactions and represented the network in Additional file 7: Figure S2. Following this initial inquiry, we looked at every individual interaction between miRNA-target and these can be seen in Additional file 7: Figure S2.

In Additional file 8: Figure S3A, the target genes for miR-23a-3p involved in PI3K-AKT signaling pathway and tight junctions are presented, and respectively emphasized in red and blue. Additional file 7: Figure S2B shows the target genes for miR-139-5p involved in Chemokine signaling pathway colored as blue, while the targeted genes involved focal adhesion colored as red. Additional file 8: Figure S3C presents the target genes for miR-141-3p that regulate the cell cycle in blue and the most frequently mutated miRNA-regulated genes in cancer in red. Additional file 8: Figure S3D presents the target genes for miR-143-5p involved in focal adhesion in blue and the most frequently mutated miRNA-regulated genes in cancer in red. Additional file 8: Figure S3E presents the target genes for miR-205-5p involved in adherent junctions in yellow, focal adhesion in green, the most frequently modified cancer pathways in blue and the most frequently mutated miRNA-regulated genes in cancer in red. In its entirety, Additional file 8: Figure S3 emphasizes the important relationship between EMT and miRNAs in terms of the frequently mutated genes or dysregulated pathways; with the chosen miRNAs, governing some aspect of cell adhesion. Multiple core regulatory processes or signaling pathways have demonstrated to be of functionally relevance to bladder cancer tumor progression, particularly in terms of invasiveness and reoccurrence. This has essential impact for bladder cancer in terms of diagnosis and/or prognosis.

### Network analysis of altered miRNA and frequent mutated genes

**Fig. 7:**
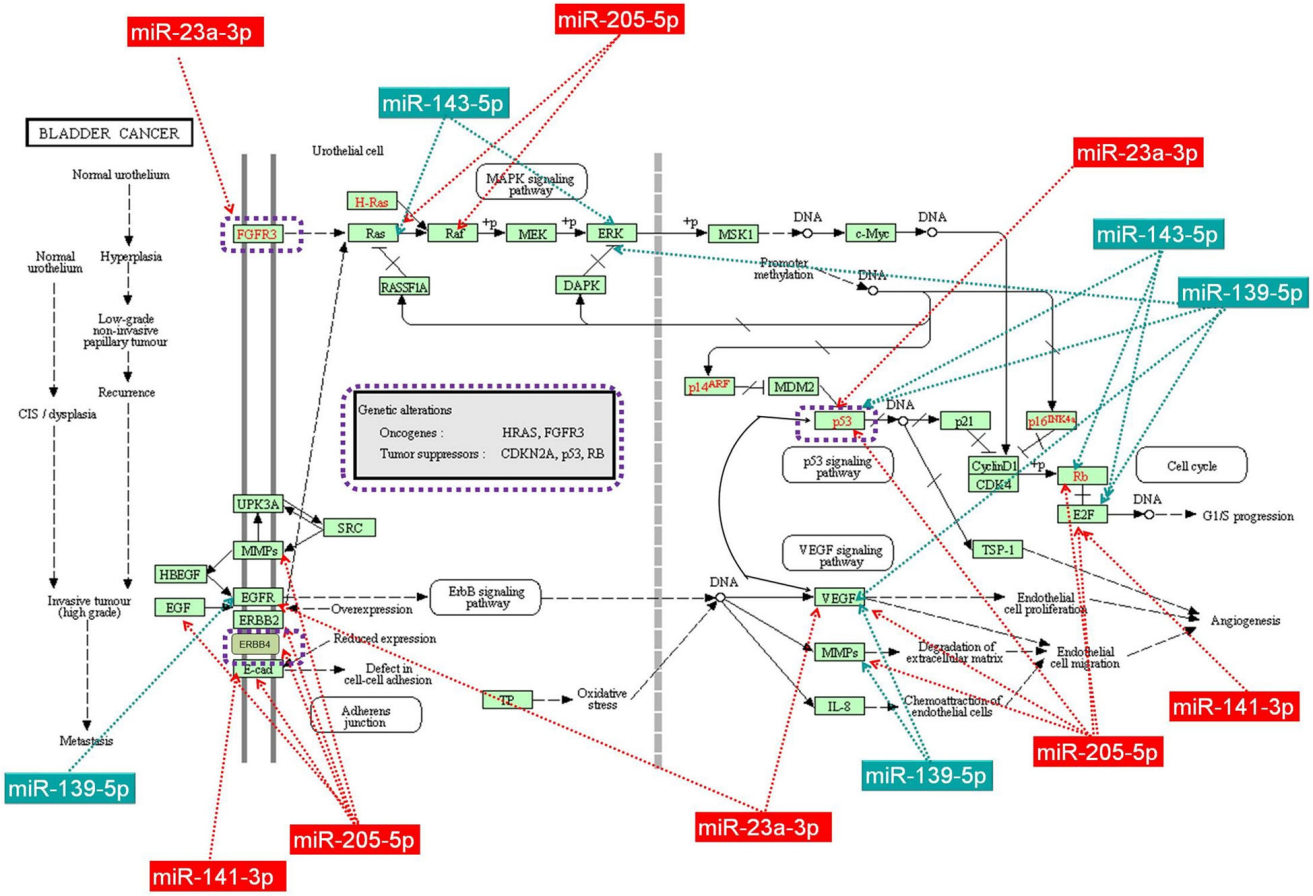
MicroRNAs downregulated (miR-139-5p and miR-143-5p) and overexpressed (miR-23a-3p, miR-141-3p, and miR-205-5p) in bladder cancer target multiple genes involved in the bladder cancer carcinogenesis network as displayed by KEEG. The red rectangles represent the upregulated miRNAs while the dark green rectangles represent the downregulated miRNAs. This Fig. was developed based on the output of KEEG database

The interconnection between mutated genes and altered miRNAs evaluated can been seen in Fig. 7. As was expected, miRNAs are not isolated transcripts. This is exemplified by the large number of miRNAs, with altered expression levels in bladder cancer, connected to the most frequently mutated genes (*TP53, FGFR3, KDR, PIK3CA* and *ATM*). Figure 7 also emphasizes the direct or indirect mode of affecting the transcriptomic pattern, which includes other miRNAs. Alterations in some genes, including *TP53, FGFR3, ATM*, *KDR* and *PIK3CA*, have been shown to trigger carcinogenesis and become intertwined with a high number of altered miRNA transcripts in bladder cancer (Additional file 8: Figure S3).

## Discussions

Bladder cancer is a very heterogeneous disease, which makes the matched/paired samples advantageous in analyzing their molecular profile. This allows a miRNAs profiling evaluation from a limited number of patients; however, it is the overlapping with TCGA data that specifically permits the identification of the most representative altered transcripts. The importance to understanding any disease is not in the specific quantifications of certain transcriptomic information (like miRNA). Rather in how this transcriptomic information relates to specific genetic alterations and their intrinsic cellular mechanisms, which affect the equilibrium therein facilitating the progression of or toward the disease.

It was identified a specific miRNA pattern in bladder cancer, from which a selected subset of individual miRNAs and their combinations were associated with overall survival rate. This study represents the basis for developing miRNA expression signatures as diagnostic tools for BC and also encourages our comprehension of the miRNA function in the initiation or progression of disease. The integrated miRNA profiling of bladder carcinomas reported here extends our knowledge based on building a comprehensive characteristic miRNAs signature with clinical importance for prognosis or diagnosis.

Our data demonstrates consistent dysregulation of miRNAs in bladder cancer. Some of the altered miRNAs are exhibited in all the cancer types like the cases of let-7 family members [14–18]. Also, miR-21 is a commonly up-regulated miRNA in human cancers, which also appears to be consistent in our case. In bladder cancer in vitro models, miR-21 is able to regulate cell proliferation and migration via its cross talk with PTEN and TP53 [19], this is one of the most important pathways related to bladder carcinogenesis [20]. A recent study confirmed the altered expression level for miR-21, miR-205 and miR-200c in bladder cancer when analyzing data generated by pairing the tissue to plasma samples [21]. As the previous authors concluded, we did find miR-205 to be specific to bladder tumor tissue and, in addition, this miRNA was consistently upregulated.

In addition, we were able to identify specific miRNAs signatures for epithelial transition to mesenchymal state (EMT). EMT is a well-known process related to tumor invasion, drug resistance and potential to be stem cell-like [22]. These EMT related transcripts particularly regulate loss of adhesion, leading to increasing cell motility which facilitates metastasis. It was demonstrated that genes *BCL2*, *CDH11*, *ZEB1/ZEB2* and *TIMP2* are targeted respectively by miR-200b/c, miR-200c and miR-200c causing this regulatory effect on adhesion and indirect regulation of EMT [23–26], also emphasis in Fig. 3d.

There appears to be an inverse relationship between the expression of the miR-200 family, particularly miR-200c and miR-141, and the expression of E-cadherin and concurrently exhibiting significant downregulation of ZEB1 expression [27]. This provides an important therapeutic target worth investigating further explicitly for bladder cancer to re-enforce the adhesion between cells and hinder EMT. Detailed evaluation of the expression level of the miR-200 family (miR-141/200c and miR-200a/200b/429) has shown frequent upregulation in BC, but are frequently downregulated in other cancer types, like renal cancer [28]. MiR-200c expression level is associated with early stage T1 bladder tumor progression [29]. More specifically, miR-200c downregulation was connected with progression to muscle invasive bladder cancer and unfavorable prognosis [29]. Other miRNAs with an altered expression level in bladder cancer involved in EMT and invasion is miR-141. In multiple other pathologies [30], not only bladder cancer [31], miR-200c and miR-141 were considered prognostic markers [32]. It should be noted that for the range of bladder cancer samples studied, the expression levels of miR-205 and miR-200c are highly disputed [22, 33, 34], often reported as both downregulated [35] and overexpressed [17].

In a recent review paper, miR-145 was presented as a frequently miRNA downregulated in bladder cancer [27, 36, 37], which was confirmed by our data. miR-143/145 is presented as clustered in the literature with tumor suppressor function in a wide range of cancers, including bladder cancer [28, 38]. We found that miR-143 had a downregulated expression in the bladder tumor tissue and that it interacts with RAS, ERK, P53, Rb or E2F (Fig. 8). Some authors argue that the downregulated miR-143 is regulated by the oncoprotein EZH2, frequently overexpressed in bladder cancer, representing an important therapeutic target not only a biomarker [39, 40].

Several studies present miR-133b to be downregulated in bladder cancer, but also in other cancers [36, 41]. It not only targets the mechanism of apoptosis and cell proliferation [42], but also migration and invasions via EGFR (epidermal growth factor receptor) and its downstream or upstream effectors [43, 44]. Another one of our downregulated transcripts is miR-139, whose expression level, interaction with MMPs (Fig. 8) and associated gene network of focal adhesion (Additional file 7: Figure S2B) are consistent with literature data [45, 46].

For bladder cancer, a number of the altered miRNAs were proven to regulate TP53 network at multiple levels [28, 50], emphasizing the importance of TP53 status. TP53 overexpression (by IHC) was proven to be associated with the presence of mutation in this gene that leads to its inactivation. In turn, inactivation of TP53 promotes tumorigenesis in cancer cells and, not coincidently, is correlated to poor survival in human tumors. A study done by Puzio-Kuter et al. (2009) demonstrated that the combined inactivation of TP53 and p10 in bladder epithelium lead to an invasive cancer characteristic in mouse models [51]. The authors claimed that the synergy of deleting both p53 and p10 is mediated by deregulation of mTOR signaling. Specifically, TP53 was observed to interact with miR-200a, miR-214, miR-513 and miR-1225. Additionally, miR-19 provides a possible link between the TP53, KDR, ERBB4 and PIK3CA. Low-grade carcinoma usually have *PIK3CA* mutation and it progresses into high grade tumor after in inactivation CDKN2A [52]. The presence of specific SNP on the level of *PIK3CA* was related with bladder cancer risk. Therefore, recognizing and targeting genetic variations of the PI3K/AKT/mTOR pathway has an important clinical implication for bladder cancer prognosis [53].

TP53 mutation alteration of the receptors tyrosine kinase pathways are observed in our investigation, which is in agreement with literature date. This pathway is activated in around 40% bladder cancer cases (*FGFR3:* > 10%, *EGFR:*> 10%, *ERBB2:*~ 10%, *ERBB3:*~ 10%, *NRAS/HRAS/KRAS:*~ 10%, *PTEN*: ~ 10%, *AKT3*:~ 10%) [54]. TCGA data revealed 58 significantly mutated genes and high incidence of several genetic pathways [52]. In our study, an important difference between the overall survival of *TP53* mutant and wild-type was confirmed; a fact established by previous studies on bladder cancers [55–57]. Literature data reports frequent alterations on the expression levels of FGFRs [7, 58], which if further clarified and has significant role in the response to therapy [59, 58]. In our case, a single exonic mutation (exonic: NM_000142.4) in 13/24(54%) was identified and determined to be pathogenic using the FATHMM web server. *FGFR3* and *TP53* have proven to have a higher incidence of mutation in our analyzed samples. Using miRnet database, we demonstrated (see Additional file 8: Figure S3.) that these genes do interact with several miRNAs that were altered in our investigation, however, the specifics of the interaction has yet to be discovered. Based on our results and using the KEGG database, in Fig. 8 we proposed interactions between the genes associated to BC carcinogenesis and our altered miRNAs expressions (▼ miR-139-5p; ▼ miR-143-5p; ▲ miR-23a-3p; ▲ miR-141-3p; ▲ miR-205-5p). In the end, it is important to highlight the complexity of the transcriptome and its interactions with the genome, especially if mutational status is added as another level of analysis.

Lastly, ERBB4 (the epidermal growth factor receptor 4) belong to the ERB family of growth factor receptor tyrosine kinases (TKI) being involved in the regulation of cell proliferation, cell differentiation, migration and invasion related in mechanism by two major pathways Ras/Raf/MAPK and PI3K/Akt/mTOR signaling [60]. The presence of this type of mutation was identified in 11 of our 23 paired samples analyzed and has a clinically important application in predicting the response to TKI inhibitors. ERBB4 mutated gene has been shown to be interconnected with the aforementioned miR-145 and miR-193a, but we identified only an intronic mutation, with no pathogenic effect.

## Conclusions

Alteration of the miRNAs expression level affects tumor molecular phenotype. These changes are detectable indirectly through the pathways with which they exhibit BC; for example, the presence of some specific mutations in key genes found in these pathways. Genes are acted upon by miRNAs where altering their expression acts on a variety of functions that impact cancer development and prognosis. More specific examples can be found with the EMT related miRNAs (miR-141b, miR-200 s or miR-205) that have an important role in the molecular mechanisms, underlying key processes related to tumorigenesis, invasion and metastasis.

To further extend the complexity of interactions, miRNAs can simultaneously target multiple components of the same signaling pathway or of multiple signaling pathways. By integrating of relevant mutations with the altered miRNA expressions, a better insight into this complex network is attained which contributes to an enhanced understanding of bladder cancer tumorigenesis, progression and recurrence. The combined understanding of molecular data facilitates the identification of important targets at cellular level that will lead to new clinical and biological strategies to manage a personalized treatment in BC [61]. Although the numbers of cases were limited in this NGS study, we were able to demonstrate the aberrations in genes disturbing different signaling pathways, particularly those relating to the regulation of EMT. Moreover, we were able to demonstrate the crosstalk among the frequently mutated genes TP53, FGFR3, PIK3CA or ATM from a representative list of the altered miRNA transcripts (presented in Fig. 6 and Figure S4), serving as therapeutic intervention points or as potential biomarkers.

## Supplementary information

**Additional file 1: Table S1.** The main altered miRNAs in the case of 23 matched paired samples (fold change±2, *p*-valued ≤0.05).

**Additional file 2: Table S2.** Demographic and histopathological characteristics of TCGA bladder cancer patient cohort.

**Additional file 3: Table S3.** The main altered miRNAs 409 tumours and 19 tumour-adjacent normal tissues retrieved from TCGA (fold change±2, *p*-valued ≤0.05).

**Additional file 4: Table S4.** miRNA with and altered expression level in high grade versus low grade bladder cancer- UMPh patient cohort.

**Additional file 5: Table S5.** miRNA with and altered expression level in high grade versus low grade bladder cancer- TCGA patient cohort.

**Additional file 6: Figure S1.** Bladder cancer survival curves showing the overall survival rate for TCGA bladder cancer patients for the most frequent mutated genes retrieved in our study, bases on mutation status.

**Additional file 7: Figure S2.** Target gene network generated using String10.5 based on the interactions from the miRTarBase database for (A) miR-23a-3p (B) miR-139-5p, (C) miR-141-3p, (D) miR-143-5p and (F) miR-205-5p.

**Additional file 8: Figure S3.** A Network analysis of the mutated genes in relation to their targeted miRNAs in bladder cancer. Schematic representation was obtained from miRnet (https://www.mirnet.ca/).

**Additional file 9:** **Table S9.** NGS sequencing data for bladder cancer.

## Data Availability

The TCGA material is public available. In the case of microarray plasma data are available on arrayexpress database (ID: E-MTAB-8356), will be public released immediately after the publication.

## Abbreviations

BC: Bladder cancer
EMT: Epithelial to mesenchymal transition
ERBB4: Epidermal growth factor receptor 4
miRNAs: MicroRNAs
TCGA: The Cancer Genome Atlas
TKI: Tyrosine kinases receptor
TN: Normal adjacent tissues
TT: Tumor tissues
TURBT: The transurethral resection of bladder tumour
UMPh: The Iuliu Hatieganu University of Medicine and Pharmacy, Cluj-Napoca, Romania
