# Supplementary material for: Connecting the dots between different networks: miRNAs associated with bladder cancer risk and progression
Source: J Exp Clin Cancer Res. 2019 Oct 29;38:433. doi: 10.1186/s13046-019-1406-6 (PMC6819535; doi:10.1186/s13046-019-1406-6)
Supplement: Supplementary file 1 — Additional file 1: Table S1. The main altered miRNAs in the case of 23 matched paired samples (fold change±2, p-valued ≤0.05). [file 13046_2019_1406_MOESM1_ESM.docx]

Table S1. The main altered miRNAs in the case of 23 matched paired samples (fold change±2, p-valued ≤0.05).

|  | **miRNA** | **Fold Change (abs)** | **p (Corr)** |
| --- | --- | --- | --- |
| 1 | hsa-miR-143-5p | -3.311618 | 0.003004 |
| 2 | hsa-miR-133b | -2.9178567 | 0.007502 |
| 3 | hsa-miR-139-5p | -2.3441553 | 0.004736 |
| 4 | hsa-miR-145-3p | -2.3268716 | 0.006756 |
| 5 | hsa-miR-548q | -2.265413 | 0.019142 |
| 6 | hsa-miR-4324 | -2.1516783 | 0.004296 |
| 7 | hsa-miR-214-3p | -2.1015332 | 0.02314 |
| 8 | hsa-miR-193a-5p | -2.083671 | 0.006262 |
| 9 | hsa-miR-141-3p | 9.791679 | 0.002445 |
| 10 | hsa-miR-1246 | 9.667706 | 0.002445 |
| 11 | hsa-miR-4284 | 9.127345 | 0.002445 |
| 12 | hsa-miR-21-5p | 8.711691 | 0.014209 |
| 13 | hsa-miR-200b-3p | 7.5837164 | 0.002445 |
| 14 | hsa-miR-720 | 7.539665 | 0.003004 |
| 15 | hsa-miR-205-5p | 6.2832446 | 0.016914 |
| 16 | hsa-miR-200c-3p | 6.1295466 | 0.00353 |
| 17 | hsa-let-7a-5p | 5.9361005 | 0.016749 |
| 18 | hsa-let-7f-5p | 5.6620364 | 0.019142 |
| 19 | hsa-miR-1275 | 5.4692826 | 0.011382 |
| 20 | hsa-miR-940 | 5.365646 | 0.016749 |
| 21 | hsa-miR-513a-5p | 5.1622524 | 0.004736 |
| 22 | hsa-miR-3162-5p | 5.021476 | 0.01868 |
| 23 | hsa-miR-200a-3p | 4.703963 | 0.003004 |
| 24 | hsa-let-7b-5p | 4.2954426 | 0.02314 |
| 25 | hsa-miR-23a-3p | 4.2150245 | 0.040333 |
| 26 | hsa-miR-4286 | 3.977593 | 0.01122 |
| 27 | hsa-miR-630 | 3.6476955 | 0.042829 |
| 28 | hsa-miR-191-3p | 3.385491 | 0.034283 |
| 29 | hsa-miR-1225-5p | 3.2804837 | 0.042829 |
| 30 | hsa-let-7g-5p | 3.2616782 | 0.034073 |
| 31 | hsa-miR-1234-3p | 3.1750631 | 0.019142 |
| 32 | hsa-miR-181b-5p | 2.9077084 | 0.018394 |
| 33 | hsa-miR-19b-3p | 2.6530588 | 0.02314 |
| 34 | hsa-miR-429 | 2.6181614 | 0.011523 |
