# Supplementary material for: Connecting the dots between different networks: miRNAs associated with bladder cancer risk and progression
Source: J Exp Clin Cancer Res. 2019 Oct 29;38:433. doi: 10.1186/s13046-019-1406-6 (PMC6819535; doi:10.1186/s13046-019-1406-6)
Supplement: Supplementary file 2 — Additional file 2: Table S2. Demographic and histopathological characteristics of TCGA bladder cancer patient cohort. [file 13046_2019_1406_MOESM2_ESM.docx]

Table S2. Demographic and histopathological characteristics of TCGA bladder cancer patient cohort

| Demographics | | Bladder cancer TCGA (n=409) |
| --- | --- | --- |
| Sex | Males | 302 |
|  | Females | 107 |
| Age | Median, Range | 69, 34 – 90 |
|  | Median, Range ♂ | 68, 34 – 90 |
|  | Median, Range ♀ | 72, 43 – 90 |
| Diagnosis subtype | Papillary | 131 |
|  | Non-Papillary | 273 |
|  | Unknown | 5 |
| TNM | T0 | 1 |
|  | T1 | 3 |
|  | T2 | 120 |
|  | T3 | 194 |
|  | T4 | 58 |
|  | Tx | 1 |
|  | T unknown | 33 |
|  | N0 | 236 |
|  | N1 | 46 |
|  | N2 | 76 |
|  | N3 | 8 |
|  | Nx | 36 |
|  | N unknown | 6 |
|  | M0 | 195 |
|  | M1 | 11 |
|  | Mx | 200 |
|  | M unknown | 3 |
| Turmor stage | I | 2 |
|  | II | 131 |
|  | III | 139 |
|  | IV | 135 |
|  | Unknown | 2 |
| Histologic Grade | High grade | 385 |
|  | Low grade | 21 |
|  | Unknown | 3 |
