# Supplementary material for: Connecting the dots between different networks: miRNAs associated with bladder cancer risk and progression
Source: J Exp Clin Cancer Res. 2019 Oct 29;38:433. doi: 10.1186/s13046-019-1406-6 (PMC6819535; doi:10.1186/s13046-019-1406-6)
Supplement: Supplementary file 3 — Additional file 3: Table S3. The main altered miRNAs 409 tumours and 19 tumour-adjacent normal tissues retrieved from TCGA (fold change±2, p-valued ≤0.05). [file 13046_2019_1406_MOESM3_ESM.docx]

Table S3. The main altered miRNAs 409 tumours and 19 tumour-adjacent normal tissues retrieved from TCGA (fold change±2, p-valued ≤0.05).

| No. | Sample | FC (abs) | p (Corr) |
| --- | --- | --- | --- |
| 1 | hsa-mir-1-2 | -10.1808 | 3.92E-13 |
| 2 | hsa-mir-133a-1 | -9.12841 | 2.65E-13 |
| 3 | hsa-mir-133b | -7.91762 | 4.22E-15 |
| 4 | hsa-mir-490 | -7.8352 | 5.89E-10 |
| 5 | hsa-mir-1247 | -6.97934 | 1.60E-07 |
| 6 | hsa-mir-143 | -5.98754 | 3.47E-13 |
| 7 | hsa-let-7c | -4.19794 | 4.44E-08 |
| 8 | hsa-mir-383 | -4.16775 | 1.33E-10 |
| 9 | hsa-mir-133a-2 | -4.01515 | 1.95E-16 |
| 10 | hsa-mir-99a | -3.66478 | 5.97E-05 |
| 11 | hsa-mir-139 | -3.61284 | 4.77E-13 |
| 12 | hsa-mir-204 | -3.29435 | 6.02E-06 |
| 13 | hsa-mir-145 | -2.97245 | 4.83E-06 |
| 14 | hsa-mir-125b-2 | -2.83934 | 3.41E-05 |
| 15 | hsa-mir-100 | -2.75649 | 1.15E-04 |
| 16 | hsa-mir-195 | -2.51276 | 3.50E-08 |
| 17 | hsa-mir-30a | -2.38777 | 2.70E-06 |
| 18 | hsa-mir-125b-1 | -2.19814 | 0.001635 |
| 19 | hsa-mir-504 | -1.97977 | 7.20E-11 |
| 20 | hsa-mir-1298 | -1.87712 | 6.26E-12 |
| 21 | hsa-mir-1258 | -1.79154 | 4.02E-11 |
| 22 | hsa-mir-210 | 47.79911 | 1.84E-23 |
| 23 | hsa-mir-141 | 20.55746 | 4.20E-20 |
| 24 | hsa-mir-183 | 18.02257 | 1.84E-23 |
| 25 | hsa-mir-205 | 16.2004 | 1.33E-10 |
| 26 | hsa-mir-200a | 14.43499 | 5.60E-14 |
| 27 | hsa-mir-429 | 13.98208 | 2.50E-13 |
| 28 | hsa-mir-200b | 12.02037 | 4.23E-12 |
| 29 | hsa-mir-182 | 11.48031 | 3.31E-19 |
| 30 | hsa-mir-96 | 11.28078 | 3.25E-22 |
| 31 | hsa-mir-200c | 11.15896 | 9.68E-14 |
| 32 | hsa-mir-18a | 9.134554 | 1.62E-22 |
| 33 | hsa-mir-20a | 8.627134 | 6.27E-23 |
| 34 | hsa-mir-203 | 8.507063 | 1.01E-05 |
| 35 | hsa-mir-93 | 8.173837 | 6.27E-23 |
| 36 | hsa-mir-130b | 8.088646 | 3.86E-21 |
| 37 | hsa-mir-934 | 7.968028 | 1.94E-06 |
| 38 | hsa-mir-425 | 7.94163 | 6.27E-23 |
| 39 | hsa-mir-767 | 7.927967 | 4.52E-04 |
| 40 | hsa-mir-31 | 7.821529 | 4.83E-06 |
| 41 | hsa-mir-19a | 7.352068 | 3.25E-22 |
| 42 | hsa-mir-1307 | 7.214008 | 3.93E-23 |
| 43 | hsa-mir-33a | 6.970275 | 6.11E-16 |
| 44 | hsa-mir-345 | 6.873473 | 1.23E-14 |
| 45 | hsa-mir-149 | 6.836433 | 6.12E-12 |
| 46 | hsa-mir-17 | 6.821994 | 3.59E-24 |
| 47 | hsa-mir-335 | 6.769179 | 6.32E-10 |
| 48 | hsa-mir-708 | 6.6876 | 3.56E-13 |
| 49 | hsa-mir-21 | 6.664092 | 1.47E-42 |
| 50 | hsa-mir-455 | 6.647373 | 2.45E-14 |
| 51 | hsa-mir-105-2 | 6.55458 | 7.35E-04 |
| 52 | hsa-mir-135b | 6.548152 | 1.33E-08 |
| 53 | hsa-mir-105-1 | 6.508137 | 7.45E-04 |
| 54 | hsa-mir-192 | 6.232041 | 5.93E-15 |
| 55 | hsa-mir-584 | 6.218042 | 4.09E-10 |
| 56 | hsa-mir-224 | 6.137013 | 2.50E-06 |
| 57 | hsa-mir-301a | 5.990325 | 9.69E-18 |
| 58 | hsa-mir-142 | 5.548953 | 7.04E-10 |
| 59 | hsa-mir-181b-1 | 5.443679 | 1.32E-18 |
| 60 | hsa-mir-19b-2 | 5.42674 | 1.86E-21 |
| 61 | hsa-mir-944 | 5.4187 | 1.82E-05 |
| 62 | hsa-mir-590 | 5.341721 | 1.81E-21 |
| 63 | hsa-mir-503 | 5.03641 | 1.69E-14 |
| 64 | hsa-mir-324 | 4.959882 | 1.65E-15 |
| 65 | hsa-mir-519a-1 | 4.949268 | 4.98E-04 |
| 66 | hsa-mir-181a-1 | 4.927864 | 8.43E-21 |
| 67 | hsa-mir-191 | 4.896875 | 1.76E-15 |
| 68 | hsa-mir-15a | 4.892503 | 1.86E-21 |
| 69 | hsa-mir-92a-1 | 4.833416 | 1.79E-15 |
| 70 | hsa-mir-185 | 4.750294 | 1.84E-23 |
| 71 | hsa-mir-193b | 4.486491 | 1.82E-12 |
| 72 | hsa-mir-181a-2 | 4.485281 | 8.68E-12 |
| 73 | hsa-mir-3613 | 4.388656 | 1.32E-20 |
| 74 | hsa-mir-32 | 4.373147 | 2.68E-17 |
| 75 | hsa-mir-483 | 4.368942 | 0.006779 |
| 76 | hsa-mir-339 | 4.321339 | 4.42E-12 |
| 77 | hsa-mir-671 | 4.267249 | 2.13E-22 |
| 78 | hsa-mir-106b | 4.251102 | 1.77E-18 |
| 79 | hsa-mir-516a-1 | 4.188225 | 0.001276 |
| 80 | hsa-mir-92a-2 | 4.171257 | 4.55E-18 |
| 81 | hsa-mir-675 | 4.120235 | 0.005673 |
| 82 | hsa-mir-103-2 | 4.078198 | 3.11E-18 |
| 83 | hsa-mir-629 | 4.073536 | 8.21E-15 |
| 84 | hsa-mir-516a-2 | 4.073307 | 0.001478 |
| 85 | hsa-mir-331 | 4.012463 | 2.61E-16 |
| 86 | hsa-mir-3065 | 3.967748 | 1.84E-07 |
| 87 | hsa-mir-106a | 3.950711 | 2.90E-09 |
| 88 | hsa-mir-454 | 3.870603 | 1.62E-20 |
| 89 | hsa-mir-148b | 3.848278 | 1.19E-23 |
| 90 | hsa-mir-940 | 3.825383 | 2.57E-10 |
| 91 | hsa-mir-3607 | 3.811367 | 5.60E-10 |
| 92 | hsa-mir-1301 | 3.743432 | 6.22E-13 |
| 93 | hsa-mir-130a | 3.738078 | 5.40E-10 |
| 94 | hsa-mir-652 | 3.737302 | 5.93E-15 |
| 95 | hsa-mir-194-2 | 3.642415 | 2.96E-09 |
| 96 | hsa-mir-301b | 3.638213 | 1.66E-09 |
| 97 | hsa-mir-34a | 3.61773 | 2.08E-13 |
| 98 | hsa-mir-181c | 3.517684 | 3.45E-12 |
| 99 | hsa-mir-20b | 3.514197 | 1.11E-04 |
| 100 | hsa-mir-16-2 | 3.500913 | 2.60E-15 |
| 101 | hsa-mir-19b-1 | 3.451145 | 2.45E-14 |
| 102 | hsa-mir-16-1 | 3.402171 | 1.80E-15 |
| 103 | hsa-mir-769 | 3.385851 | 7.99E-14 |
| 104 | hsa-mir-194-1 | 3.379833 | 3.34E-08 |
| 105 | hsa-mir-197 | 3.379622 | 8.83E-12 |
| 106 | hsa-mir-423 | 3.374611 | 1.44E-15 |
| 107 | hsa-mir-484 | 3.357598 | 1.01E-13 |
| 108 | hsa-mir-519a-2 | 3.350502 | 0.003028 |
| 109 | hsa-mir-576 | 3.299305 | 9.91E-15 |
| 110 | hsa-mir-34c | 3.260023 | 6.29E-07 |
| 111 | hsa-mir-501 | 3.259921 | 1.49E-09 |
| 112 | hsa-mir-874 | 3.245014 | 3.26E-10 |
| 113 | hsa-mir-589 | 3.229229 | 1.02E-12 |
| 114 | hsa-mir-196b | 3.223283 | 5.22E-08 |
| 115 | hsa-mir-877 | 3.221214 | 1.13E-10 |
| 116 | hsa-mir-744 | 3.21565 | 9.69E-10 |
| 117 | hsa-mir-146b | 3.182257 | 2.52E-06 |
| 118 | hsa-mir-660 | 3.179292 | 1.86E-11 |
| 119 | hsa-mir-1180 | 3.173948 | 3.36E-08 |
| 120 | hsa-mir-942 | 3.16883 | 4.16E-11 |
| 121 | hsa-mir-15b | 3.119666 | 5.12E-13 |
| 122 | hsa-mir-29b-1 | 3.11547 | 5.13E-09 |
| 123 | hsa-mir-27a | 3.113037 | 6.13E-12 |
| 124 | hsa-mir-181b-2 | 3.102665 | 1.39E-08 |
| 125 | hsa-mir-7-1 | 3.027722 | 3.92E-10 |
| 126 | hsa-mir-29b-2 | 3.026889 | 1.18E-08 |
| 127 | hsa-mir-3200 | 2.988896 | 1.83E-05 |
| 128 | hsa-mir-126 | 2.968595 | 1.19E-09 |
| 129 | hsa-mir-424 | 2.967155 | 1.72E-09 |
| 130 | hsa-mir-1306 | 2.960405 | 2.16E-09 |
| 131 | hsa-mir-128-2 | 2.957991 | 1.79E-11 |
| 132 | hsa-mir-526b | 2.952799 | 0.00861 |
| 133 | hsa-mir-128-1 | 2.951208 | 5.77E-13 |
| 134 | hsa-mir-532 | 2.951001 | 8.43E-11 |
| 135 | hsa-mir-522 | 2.891303 | 0.006832 |
| 136 | hsa-mir-196a-1 | 2.857866 | 0.007563 |
| 137 | hsa-mir-3615 | 2.853323 | 4.09E-12 |
| 138 | hsa-mir-937 | 2.835189 | 5.28E-09 |
| 139 | hsa-mir-3647 | 2.819072 | 1.77E-09 |
| 140 | hsa-mir-33b | 2.805677 | 8.11E-08 |
| 141 | hsa-mir-3648 | 2.785402 | 3.48E-06 |
| 142 | hsa-mir-342 | 2.771421 | 8.03E-07 |
| 143 | hsa-mir-188 | 2.765585 | 1.86E-09 |
| 144 | hsa-mir-3614 | 2.763989 | 2.38E-06 |
| 145 | hsa-mir-186 | 2.758845 | 2.69E-14 |
| 146 | hsa-mir-450a-1 | 2.749792 | 1.41E-10 |
| 147 | hsa-mir-500a | 2.728937 | 3.91E-08 |
| 148 | hsa-mir-450b | 2.727696 | 4.98E-09 |
| 149 | hsa-mir-651 | 2.724329 | 1.61E-10 |
| 150 | hsa-mir-103-1 | 2.713872 | 1.14E-12 |
| 151 | hsa-mir-505 | 2.674221 | 4.44E-09 |
| 152 | hsa-mir-4326 | 2.651254 | 1.59E-04 |
| 153 | hsa-mir-3913-1 | 2.637072 | 5.95E-08 |
| 154 | hsa-mir-215 | 2.630268 | 0.001382 |
| 155 | hsa-mir-452 | 2.6213 | 0.004711 |
| 156 | hsa-mir-450a-2 | 2.571304 | 1.04E-09 |
| 157 | hsa-mir-25 | 2.570096 | 3.86E-10 |
| 158 | hsa-mir-512-2 | 2.569766 | 0.013658 |
| 159 | hsa-mir-512-1 | 2.546622 | 0.015359 |
| 160 | hsa-mir-520a | 2.522855 | 0.021199 |
| 161 | hsa-mir-362 | 2.515949 | 2.14E-07 |
| 162 | hsa-mir-181d | 2.500102 | 4.57E-06 |
| 163 | hsa-mir-107 | 2.470265 | 4.02E-11 |
| 164 | hsa-mir-1976 | 2.459251 | 4.53E-11 |
| 165 | hsa-mir-3653 | 2.430528 | 1.82E-06 |
| 166 | hsa-mir-2355 | 2.428962 | 5.46E-06 |
| 167 | hsa-mir-151 | 2.418136 | 2.17E-07 |
| 168 | hsa-mir-148a | 2.408819 | 1.52E-05 |
| 169 | hsa-mir-3074 | 2.401057 | 6.26E-05 |
| 170 | hsa-mir-520b | 2.386708 | 0.015347 |
| 171 | hsa-let-7g | 2.38028 | 1.09E-12 |
| 172 | hsa-mir-653 | 2.375305 | 7.39E-05 |
| 173 | hsa-mir-551b | 2.368349 | 0.008354 |
| 174 | hsa-mir-550a-1 | 2.356205 | 1.67E-09 |
| 175 | hsa-mir-3934 | 2.352554 | 1.83E-08 |
| 176 | hsa-mir-22 | 2.323619 | 2.93E-13 |
| 177 | hsa-mir-155 | 2.298176 | 0.00336 |
| 178 | hsa-mir-363 | 2.28156 | 0.003813 |
| 179 | hsa-mir-625 | 2.273295 | 1.22E-05 |
| 180 | hsa-mir-542 | 2.266618 | 8.20E-07 |
| 181 | hsa-mir-92b | 2.253277 | 2.30E-05 |
| 182 | hsa-mir-219-1 | 2.240202 | 9.62E-07 |
| 183 | hsa-mir-500b | 2.239965 | 3.49E-07 |
| 184 | hsa-mir-502 | 2.239003 | 1.12E-08 |
| 185 | hsa-mir-10a | 2.224307 | 0.011793 |
| 186 | hsa-mir-146a | 2.204635 | 0.00697 |
| 187 | hsa-mir-138-1 | 2.204218 | 0.001905 |
| 188 | hsa-mir-518c | 2.193392 | 0.030631 |
| 189 | hsa-mir-3677 | 2.192467 | 9.28E-05 |
| 190 | hsa-mir-518b | 2.180059 | 0.034652 |
| 191 | hsa-mir-98 | 2.149868 | 4.35E-08 |
| 192 | hsa-mir-525 | 2.146076 | 0.046133 |
| 193 | hsa-mir-147b | 2.141809 | 3.78E-05 |
| 194 | hsa-mir-421 | 2.130324 | 3.87E-07 |
| 195 | hsa-mir-323b | 2.116933 | 2.52E-05 |
| 196 | hsa-mir-320b-2 | 2.106082 | 2.80E-06 |
| 197 | hsa-mir-3676 | 2.105907 | 5.70E-05 |
| 198 | hsa-mir-34b | 2.072184 | 7.00E-05 |
| 199 | hsa-mir-330 | 2.06587 | 7.88E-06 |
| 200 | hsa-mir-615 | 2.055377 | 4.34E-04 |
| 201 | hsa-let-7d | 2.052572 | 2.87E-08 |
| 202 | hsa-mir-361 | 2.048091 | 7.02E-12 |
| 203 | hsa-mir-153-2 | 2.047389 | 7.54E-05 |
| 204 | hsa-mir-616 | 2.039544 | 4.02E-07 |
| 205 | hsa-mir-577 | 2.038096 | 0.01481 |
| 206 | hsa-mir-24-2 | 2.027936 | 3.24E-07 |
| 207 | hsa-mir-1277 | 2.026419 | 6.72E-08 |
| 208 | hsa-mir-23a | 2.010114 | 6.21E-08 |
| 209 | hsa-mir-766 | 1.977682 | 6.77E-05 |
| 210 | hsa-mir-3651 | 1.977429 | 1.08E-04 |
| 211 | hsa-mir-138-2 | 1.974145 | 0.002263 |
| 212 | hsa-mir-136 | 1.971195 | 0.001905 |
| 213 | hsa-mir-1296 | 1.96165 | 1.82E-04 |
| 214 | hsa-mir-409 | 1.953118 | 5.63E-04 |
| 215 | hsa-mir-3687 | 1.935404 | 1.80E-04 |
| 216 | hsa-mir-1910 | 1.934957 | 7.39E-05 |
| 217 | hsa-mir-550a-2 | 1.925471 | 4.33E-06 |
| 218 | hsa-mir-3127 | 1.912391 | 2.04E-04 |
| 219 | hsa-mir-296 | 1.899065 | 0.020251 |
| 220 | hsa-mir-26b | 1.889399 | 5.16E-06 |
| 221 | hsa-mir-627 | 1.875238 | 5.39E-07 |
| 222 | hsa-mir-1226 | 1.860954 | 9.91E-05 |
| 223 | hsa-mir-760 | 1.838574 | 7.51E-04 |
| 224 | hsa-mir-222 | 1.831054 | 0.006084 |
| 225 | hsa-mir-1266 | 1.820285 | 0.013928 |
| 226 | hsa-mir-1293 | 1.812285 | 0.007558 |
| 227 | hsa-let-7e | 1.807524 | 2.65E-04 |
| 228 | hsa-mir-1287 | 1.80015 | 0.002263 |
| 229 | hsa-mir-1304 | 1.796323 | 0.001929 |
| 230 | hsa-mir-642a | 1.780658 | 2.49E-04 |
| 231 | hsa-mir-99b | 1.7571 | 0.001203 |
| 232 | hsa-mir-3170 | 1.756659 | 5.70E-05 |
| 233 | hsa-mir-511-2 | 1.740775 | 0.012542 |
| 234 | hsa-mir-30b | 1.74046 | 5.20E-04 |
| 235 | hsa-mir-1270-1 | 1.714546 | 0.002218 |
| 236 | hsa-mir-628 | 1.713461 | 1.58E-05 |
| 237 | hsa-mir-3928 | 1.687706 | 3.59E-05 |
| 238 | hsa-mir-939 | 1.677718 | 4.20E-06 |
| 239 | hsa-mir-1270-2 | 1.677318 | 0.003705 |
| 240 | hsa-mir-1283-2 | 1.668263 | 0.047166 |
| 241 | hsa-mir-1229 | 1.666554 | 1.11E-04 |
| 242 | hsa-mir-30c-1 | 1.632313 | 1.13E-05 |
| 243 | hsa-mir-1254 | 1.626387 | 8.43E-05 |
| 244 | hsa-mir-624 | 1.615197 | 2.47E-05 |
| 245 | hsa-mir-2277 | 1.611768 | 1.63E-05 |
| 246 | hsa-mir-2110 | 1.608778 | 1.02E-04 |
| 247 | hsa-mir-26a-1 | 1.60732 | 2.64E-05 |
| 248 | hsa-mir-18b | 1.603262 | 0.002351 |
| 249 | hsa-mir-125a | 1.597457 | 0.003577 |
| 250 | hsa-mir-30d | 1.593517 | 0.009258 |
| 251 | hsa-mir-570 | 1.591942 | 7.33E-05 |
| 252 | hsa-mir-323 | 1.576883 | 0.035881 |
| 253 | hsa-mir-3944 | 1.564206 | 6.50E-04 |
| 254 | hsa-let-7a-3 | 1.545749 | 6.56E-05 |
| 255 | hsa-let-7a-1 | 1.543927 | 7.05E-05 |
| 256 | hsa-let-7a-2 | 1.540744 | 7.80E-05 |
| 257 | hsa-mir-24-1 | 1.537199 | 0.002351 |
| 258 | hsa-let-7b | 1.524591 | 0.004533 |
| 259 | hsa-mir-3682 | 1.524116 | 1.86E-04 |
| 260 | hsa-mir-579 | 1.523776 | 1.02E-04 |
| 261 | hsa-mir-7-3 | 1.518827 | 0.002931 |
| 262 | hsa-mir-556 | 1.517885 | 8.96E-04 |
| 263 | hsa-mir-659 | 1.516946 | 1.21E-04 |
| 264 | hsa-let-7f-1 | 1.516938 | 1.43E-04 |
| 265 | hsa-mir-193a | 1.514723 | 0.041842 |
