# Supplementary material for: Connecting the dots between different networks: miRNAs associated with bladder cancer risk and progression
Source: J Exp Clin Cancer Res. 2019 Oct 29;38:433. doi: 10.1186/s13046-019-1406-6 (PMC6819535; doi:10.1186/s13046-019-1406-6)
Supplement: Supplementary file 4 — Additional file 4: Table S4. miRNA with and altered expression level in high grade versus low grade bladder cancer- UMPh patient cohort. [file 13046_2019_1406_MOESM4_ESM.docx]

Table S4. miRNA with and altered expression level in high grade versus low grade bladder cancer- UMPh patient cohort

| No. | Sample | FC (abs) | p (Corr) |
| --- | --- | --- | --- |
| 1 | hsa-miR-145-5p | -8,33289 | 0,012051 |
| 2 | hsa-miR-125b-5p | -5,21181 | 0,049409 |
| 3 | hsa-miR-23b-3p | -4,69531 | 0,040649 |
| 4 | hsa-miR-1234-3p | -3,77505 | 0,045164 |
| 5 | hsa-miR-10a-5p | -3,74149 | 0,043455 |
| 6 | hsa-miR-595 | -3,55984 | 0,041744 |
| 7 | hsa-miR-605 | -1,66158 | 0,042804 |
| 8 | hsa-miR-19a-3p | 3,094247 | 0,047899 |
| 9 | hsa-miR-512-3p | 2,646375 | 0,030235 |
| 10 | hsa-miR-17-3p | 2,410736 | 0,022184 |
| 11 | hsa-miR-431-3p | 2,162818 | 0,019487 |
| 12 | hsa-miR-500a-3p | 1,88038 | 0,013213 |
