# Supplementary material for: Connecting the dots between different networks: miRNAs associated with bladder cancer risk and progression
Source: J Exp Clin Cancer Res. 2019 Oct 29;38:433. doi: 10.1186/s13046-019-1406-6 (PMC6819535; doi:10.1186/s13046-019-1406-6)
Supplement: Supplementary file 5 — Additional file 5: Table S5. miRNA with and altered expression level in high grade versus low grade bladder cancer- TCGA patient cohort. [file 13046_2019_1406_MOESM5_ESM.docx]

Table S5. miRNA with and altered expression level in high grade versus low grade bladder cancer- TCGA patient cohort

| No. | Sample | FC (abs) | p (Corr) |
| --- | --- | --- | --- |
| 1 | hsa-miR-1-2 | -10,1808 | 3,92E-13 |
| 2 | hsa-miR-133a-1 | -9,12841 | 2,65E-13 |
| 3 | hsa-miR-133b | -7,91762 | 4,22E-15 |
| 4 | hsa-miR-490 | -7,8352 | 5,89E-10 |
| 5 | hsa-miR-1247 | -6,97934 | 1,60E-07 |
| 6 | hsa-miR-143 | -5,98754 | 3,47E-13 |
| 7 | hsa-let-7c | -4,19794 | 4,44E-08 |
| 8 | hsa-miR-383 | -4,16775 | 1,33E-10 |
| 9 | hsa-miR-133a-2 | -4,01515 | 1,95E-16 |
| 10 | hsa-miR-99a | -3,66478 | 5,97E-05 |
| 11 | hsa-miR-139 | -3,61284 | 4,77E-13 |
| 12 | hsa-miR-204 | -3,29435 | 6,02E-06 |
| 13 | hsa-miR-145 | -2,97245 | 4,83E-06 |
| 14 | hsa-miR-125b-2 | -2,83934 | 3,41E-05 |
| 15 | hsa-miR-100 | -2,75649 | 1,15E-04 |
| 16 | hsa-miR-195 | -2,51276 | 3,50E-08 |
| 17 | hsa-miR-30a | -2,38777 | 2,70E-06 |
| 18 | hsa-miR-125b-1 | -2,19814 | 0,001635407 |
| 19 | hsa-miR-210 | 47,79911 | 1,84E-23 |
| 20 | hsa-miR-141 | 20,55746 | 4,20E-20 |
| 21 | hsa-miR-183 | 18,02257 | 1,84E-23 |
| 22 | hsa-miR-205 | 16,2004 | 1,33E-10 |
| 23 | hsa-miR-200a | 14,43499 | 5,60E-14 |
| 24 | hsa-miR-429 | 13,98208 | 2,50E-13 |
| 25 | hsa-miR-200b | 12,02037 | 4,23E-12 |
| 26 | hsa-miR-182 | 11,48031 | 3,31E-19 |
| 27 | hsa-miR-96 | 11,28078 | 3,25E-22 |
| 28 | hsa-miR-200c | 11,15896 | 9,68E-14 |
| 29 | hsa-miR-18a | 9,134554 | 1,62E-22 |
| 30 | hsa-miR-20a | 8,627134 | 6,27E-23 |
| 31 | hsa-miR-203 | 8,507063 | 1,01E-05 |
| 32 | hsa-miR-93 | 8,173837 | 6,27E-23 |
| 33 | hsa-miR-130b | 8,088646 | 3,86E-21 |
| 34 | hsa-miR-934 | 7,968028 | 1,94E-06 |
| 35 | hsa-miR-425 | 7,94163 | 6,27E-23 |
| 36 | hsa-miR-767 | 7,927967 | 4,52E-04 |
| 37 | hsa-miR-31 | 7,821529 | 4,83E-06 |
| 38 | hsa-miR-19a | 7,352068 | 3,25E-22 |
| 39 | hsa-miR-1307 | 7,214008 | 3,93E-23 |
| 40 | hsa-miR-33a | 6,970275 | 6,11E-16 |
| 41 | hsa-miR-345 | 6,873473 | 1,23E-14 |
| 42 | hsa-miR-149 | 6,836433 | 6,12E-12 |
| 43 | hsa-miR-17 | 6,821994 | 3,59E-24 |
| 44 | hsa-miR-335 | 6,769179 | 6,32E-10 |
| 45 | hsa-miR-708 | 6,6876 | 3,56E-13 |
| 46 | hsa-miR-21 | 6,664092 | 1,47E-42 |
| 47 | hsa-miR-455 | 6,647373 | 2,45E-14 |
| 48 | hsa-miR-105-2 | 6,55458 | 7,35E-04 |
| 49 | hsa-miR-135b | 6,548152 | 1,33E-08 |
| 50 | hsa-miR-105-1 | 6,508137 | 7,45E-04 |
| 51 | hsa-miR-192 | 6,232041 | 5,93E-15 |
| 52 | hsa-miR-584 | 6,218042 | 4,09E-10 |
| 53 | hsa-miR-224 | 6,137013 | 2,50E-06 |
| 54 | hsa-miR-301a | 5,990325 | 9,69E-18 |
| 55 | hsa-miR-142 | 5,548953 | 7,04E-10 |
| 56 | hsa-miR-181b-1 | 5,443679 | 1,32E-18 |
| 57 | hsa-miR-19b-2 | 5,42674 | 1,86E-21 |
| 58 | hsa-miR-944 | 5,4187 | 1,82E-05 |
| 59 | hsa-miR-590 | 5,341721 | 1,81E-21 |
| 60 | hsa-miR-503 | 5,03641 | 1,69E-14 |
| 61 | hsa-miR-324 | 4,959882 | 1,65E-15 |
| 62 | hsa-miR-519a-1 | 4,949268 | 4,98E-04 |
| 63 | hsa-miR-181a-1 | 4,927864 | 8,43E-21 |
| 64 | hsa-miR-191 | 4,896875 | 1,76E-15 |
| 65 | hsa-miR-15a | 4,892503 | 1,86E-21 |
| 66 | hsa-miR-92a-1 | 4,833416 | 1,79E-15 |
| 67 | hsa-miR-185 | 4,750294 | 1,84E-23 |
| 68 | hsa-miR-193b | 4,486491 | 1,82E-12 |
| 69 | hsa-miR-181a-2 | 4,485281 | 8,68E-12 |
| 70 | hsa-miR-3613 | 4,388656 | 1,32E-20 |
| 71 | hsa-miR-32 | 4,373147 | 2,68E-17 |
| 72 | hsa-miR-483 | 4,368942 | 0,006779311 |
| 73 | hsa-miR-339 | 4,321339 | 4,42E-12 |
| 74 | hsa-miR-671 | 4,267249 | 2,13E-22 |
| 75 | hsa-miR-106b | 4,251102 | 1,77E-18 |
| 76 | hsa-miR-516a-1 | 4,188225 | 0,001275671 |
| 77 | hsa-miR-92a-2 | 4,171257 | 4,55E-18 |
| 78 | hsa-miR-675 | 4,120235 | 0,005673001 |
| 79 | hsa-miR-103-2 | 4,078198 | 3,11E-18 |
| 80 | hsa-miR-629 | 4,073536 | 8,21E-15 |
| 81 | hsa-miR-516a-2 | 4,073307 | 0,001478362 |
| 82 | hsa-miR-331 | 4,012463 | 2,61E-16 |
| 83 | hsa-miR-3065 | 3,967748 | 1,84E-07 |
| 84 | hsa-miR-106a | 3,950711 | 2,90E-09 |
| 85 | hsa-miR-454 | 3,870603 | 1,62E-20 |
| 86 | hsa-miR-148b | 3,848278 | 1,19E-23 |
| 87 | hsa-miR-940 | 3,825383 | 2,57E-10 |
| 88 | hsa-miR-3607 | 3,811367 | 5,60E-10 |
| 89 | hsa-miR-1301 | 3,743432 | 6,22E-13 |
| 90 | hsa-miR-130a | 3,738078 | 5,40E-10 |
| 91 | hsa-miR-652 | 3,737302 | 5,93E-15 |
| 92 | hsa-miR-194-2 | 3,642415 | 2,96E-09 |
| 93 | hsa-miR-301b | 3,638213 | 1,66E-09 |
| 94 | hsa-miR-34a | 3,61773 | 2,08E-13 |
| 95 | hsa-miR-181c | 3,517684 | 3,45E-12 |
| 96 | hsa-miR-20b | 3,514197 | 1,11E-04 |
| 97 | hsa-miR-16-2 | 3,500913 | 2,60E-15 |
| 98 | hsa-miR-19b-1 | 3,451145 | 2,45E-14 |
| 99 | hsa-miR-16-1 | 3,402171 | 1,80E-15 |
| 100 | hsa-miR-769 | 3,385851 | 7,99E-14 |
| 101 | hsa-miR-194-1 | 3,379833 | 3,34E-08 |
| 102 | hsa-miR-197 | 3,379622 | 8,83E-12 |
| 103 | hsa-miR-423 | 3,374611 | 1,44E-15 |
| 104 | hsa-miR-484 | 3,357598 | 1,01E-13 |
| 105 | hsa-miR-519a-2 | 3,350502 | 0,003028125 |
| 106 | hsa-miR-576 | 3,299305 | 9,91E-15 |
| 107 | hsa-miR-34c | 3,260023 | 6,29E-07 |
| 108 | hsa-miR-501 | 3,259921 | 1,49E-09 |
| 109 | hsa-miR-874 | 3,245014 | 3,26E-10 |
| 110 | hsa-miR-589 | 3,229229 | 1,02E-12 |
| 111 | hsa-miR-196b | 3,223283 | 5,22E-08 |
| 112 | hsa-miR-877 | 3,221214 | 1,13E-10 |
| 113 | hsa-miR-744 | 3,21565 | 9,69E-10 |
| 114 | hsa-miR-146b | 3,182257 | 2,52E-06 |
| 115 | hsa-miR-660 | 3,179292 | 1,86E-11 |
| 116 | hsa-miR-1180 | 3,173948 | 3,36E-08 |
| 117 | hsa-miR-942 | 3,16883 | 4,16E-11 |
| 118 | hsa-miR-15b | 3,119666 | 5,12E-13 |
| 119 | hsa-miR-29b-1 | 3,11547 | 5,13E-09 |
| 120 | hsa-miR-27a | 3,113037 | 6,13E-12 |
| 121 | hsa-miR-181b-2 | 3,102665 | 1,39E-08 |
| 122 | hsa-miR-7-1 | 3,027722 | 3,92E-10 |
| 123 | hsa-miR-29b-2 | 3,026889 | 1,18E-08 |
| 124 | hsa-miR-3200 | 2,988896 | 1,83E-05 |
| 125 | hsa-miR-126 | 2,968595 | 1,19E-09 |
| 126 | hsa-miR-424 | 2,967155 | 1,72E-09 |
| 127 | hsa-miR-1306 | 2,960405 | 2,16E-09 |
| 128 | hsa-miR-128-2 | 2,957991 | 1,79E-11 |
| 129 | hsa-miR-526b | 2,952799 | 0,00860978 |
| 130 | hsa-miR-128-1 | 2,951208 | 5,77E-13 |
| 131 | hsa-miR-532 | 2,951001 | 8,43E-11 |
| 132 | hsa-miR-522 | 2,891303 | 0,006832067 |
| 133 | hsa-miR-196a-1 | 2,857866 | 0,007563417 |
| 134 | hsa-miR-3615 | 2,853323 | 4,09E-12 |
| 135 | hsa-miR-937 | 2,835189 | 5,28E-09 |
| 136 | hsa-miR-3647 | 2,819072 | 1,77E-09 |
| 137 | hsa-miR-33b | 2,805677 | 8,11E-08 |
| 138 | hsa-miR-3648 | 2,785402 | 3,48E-06 |
| 139 | hsa-miR-342 | 2,771421 | 8,03E-07 |
| 140 | hsa-miR-188 | 2,765585 | 1,86E-09 |
| 141 | hsa-miR-3614 | 2,763989 | 2,38E-06 |
| 142 | hsa-miR-186 | 2,758845 | 2,69E-14 |
| 143 | hsa-miR-450a-1 | 2,749792 | 1,41E-10 |
| 144 | hsa-miR-500a | 2,728937 | 3,91E-08 |
| 145 | hsa-miR-450b | 2,727696 | 4,98E-09 |
| 146 | hsa-miR-651 | 2,724329 | 1,61E-10 |
| 147 | hsa-miR-103-1 | 2,713872 | 1,14E-12 |
| 148 | hsa-miR-505 | 2,674221 | 4,44E-09 |
| 149 | hsa-miR-4326 | 2,651254 | 1,59E-04 |
| 150 | hsa-miR-3913-1 | 2,637072 | 5,95E-08 |
| 151 | hsa-miR-215 | 2,630268 | 0,00138231 |
| 152 | hsa-miR-452 | 2,6213 | 0,004711176 |
| 153 | hsa-miR-450a-2 | 2,571304 | 1,04E-09 |
| 154 | hsa-miR-25 | 2,570096 | 3,86E-10 |
| 155 | hsa-miR-512-2 | 2,569766 | 0,013658368 |
| 156 | hsa-miR-512-1 | 2,546622 | 0,015358783 |
| 157 | hsa-miR-520a | 2,522855 | 0,021198802 |
| 158 | hsa-miR-362 | 2,515949 | 2,14E-07 |
| 159 | hsa-miR-181d | 2,500102 | 4,57E-06 |
| 160 | hsa-miR-107 | 2,470265 | 4,02E-11 |
| 161 | hsa-miR-1976 | 2,459251 | 4,53E-11 |
| 162 | hsa-miR-3653 | 2,430528 | 1,82E-06 |
| 163 | hsa-miR-2355 | 2,428962 | 5,46E-06 |
| 164 | hsa-miR-151 | 2,418136 | 2,17E-07 |
| 165 | hsa-miR-148a | 2,408819 | 1,52E-05 |
| 166 | hsa-miR-3074 | 2,401057 | 6,26E-05 |
| 167 | hsa-miR-520b | 2,386708 | 0,01534731 |
| 168 | hsa-let-7g | 2,38028 | 1,09E-12 |
| 169 | hsa-miR-653 | 2,375305 | 7,39E-05 |
| 170 | hsa-miR-551b | 2,368349 | 0,00835416 |
| 171 | hsa-miR-550a-1 | 2,356205 | 1,67E-09 |
| 172 | hsa-miR-3934 | 2,352554 | 1,83E-08 |
| 173 | hsa-miR-22 | 2,323619 | 2,93E-13 |
| 174 | hsa-miR-155 | 2,298176 | 0,003359886 |
| 175 | hsa-miR-363 | 2,28156 | 0,003812586 |
| 176 | hsa-miR-625 | 2,273295 | 1,22E-05 |
| 177 | hsa-miR-542 | 2,266618 | 8,20E-07 |
| 178 | hsa-miR-92b | 2,253277 | 2,30E-05 |
| 179 | hsa-miR-219-1 | 2,240202 | 9,62E-07 |
| 180 | hsa-miR-500b | 2,239965 | 3,49E-07 |
| 181 | hsa-miR-502 | 2,239003 | 1,12E-08 |
| 182 | hsa-miR-10a | 2,224307 | 0,011793216 |
| 183 | hsa-miR-146a | 2,204635 | 0,006969813 |
| 184 | hsa-miR-138-1 | 2,204218 | 0,001905033 |
| 185 | hsa-miR-518c | 2,193392 | 0,03063051 |
| 186 | hsa-miR-3677 | 2,192467 | 9,28E-05 |
| 187 | hsa-miR-518b | 2,180059 | 0,034651916 |
| 188 | hsa-miR-98 | 2,149868 | 4,35E-08 |
| 189 | hsa-miR-525 | 2,146076 | 0,04613273 |
| 190 | hsa-miR-147b | 2,141809 | 3,78E-05 |
| 191 | hsa-miR-421 | 2,130324 | 3,87E-07 |
| 192 | hsa-miR-323b | 2,116933 | 2,52E-05 |
| 193 | hsa-miR-320b-2 | 2,106082 | 2,80E-06 |
| 194 | hsa-miR-3676 | 2,105907 | 5,70E-05 |
| 195 | hsa-miR-34b | 2,072184 | 7,00E-05 |
| 196 | hsa-miR-330 | 2,06587 | 7,88E-06 |
| 197 | hsa-miR-615 | 2,055377 | 4,34E-04 |
| 198 | hsa-let-7d | 2,052572 | 2,87E-08 |
| 199 | hsa-miR-361 | 2,048091 | 7,02E-12 |
| 200 | hsa-miR-153-2 | 2,047389 | 7,54E-05 |
| 201 | hsa-miR-616 | 2,039544 | 4,02E-07 |
| 202 | hsa-miR-577 | 2,038096 | 0,014809765 |
| 203 | hsa-miR-24-2 | 2,027936 | 3,24E-07 |
| 204 | hsa-miR-1277 | 2,026419 | 6,72E-08 |
| 205 | hsa-miR-23a | 2,010114 | 6,21E-08 |
