# Supplementary figures and images for: Connecting the dots between different networks: miRNAs associated with bladder cancer risk and progression
Source: J Exp Clin Cancer Res. 2019 Oct 29;38:433. doi: 10.1186/s13046-019-1406-6 (PMC6819535; doi:10.1186/s13046-019-1406-6)

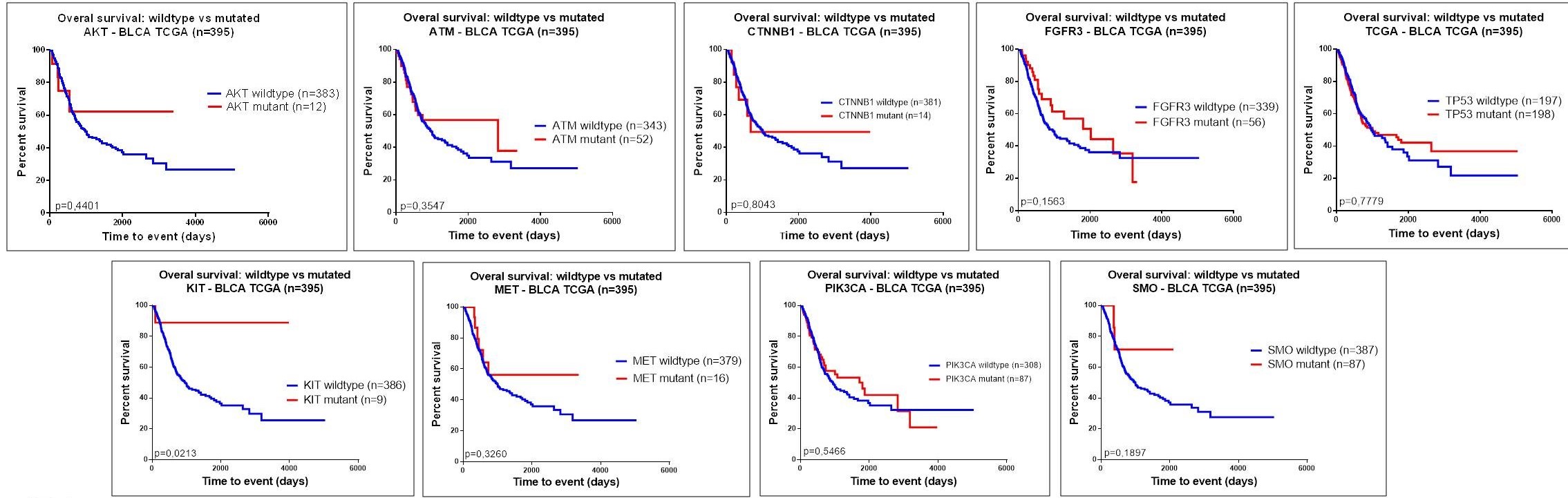

Supplement: Supplementary file 6 — Additional file 6: Figure S1. Bladder cancer survival curves showing the overall survival rate for TCGA bladder cancer patients for the most frequent mutated genes retrieved in our study, bases on mutation status. [file 13046_2019_1406_MOESM6_ESM.jpg]

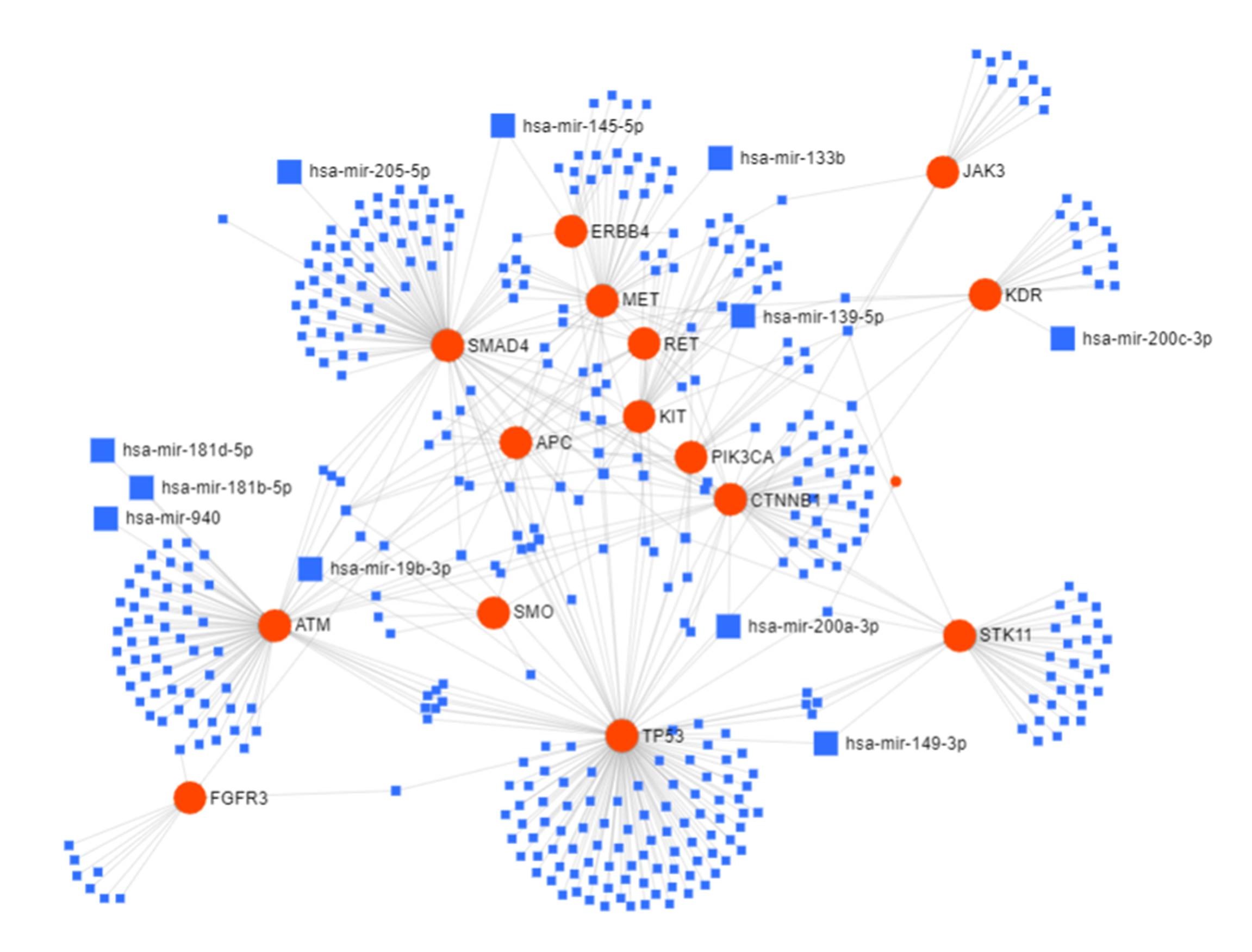

Supplement: Supplementary file 7 — Additional file 7: Figure S2. Target gene network generated using String10.5 based on the interactions from the miRTarBase database for (A) miR-23a-3p (B) miR-139-5p, (C) miR-141-3p, (D) miR-143-5p and (F) miR-205-5p. [file 13046_2019_1406_MOESM7_ESM.jpg]

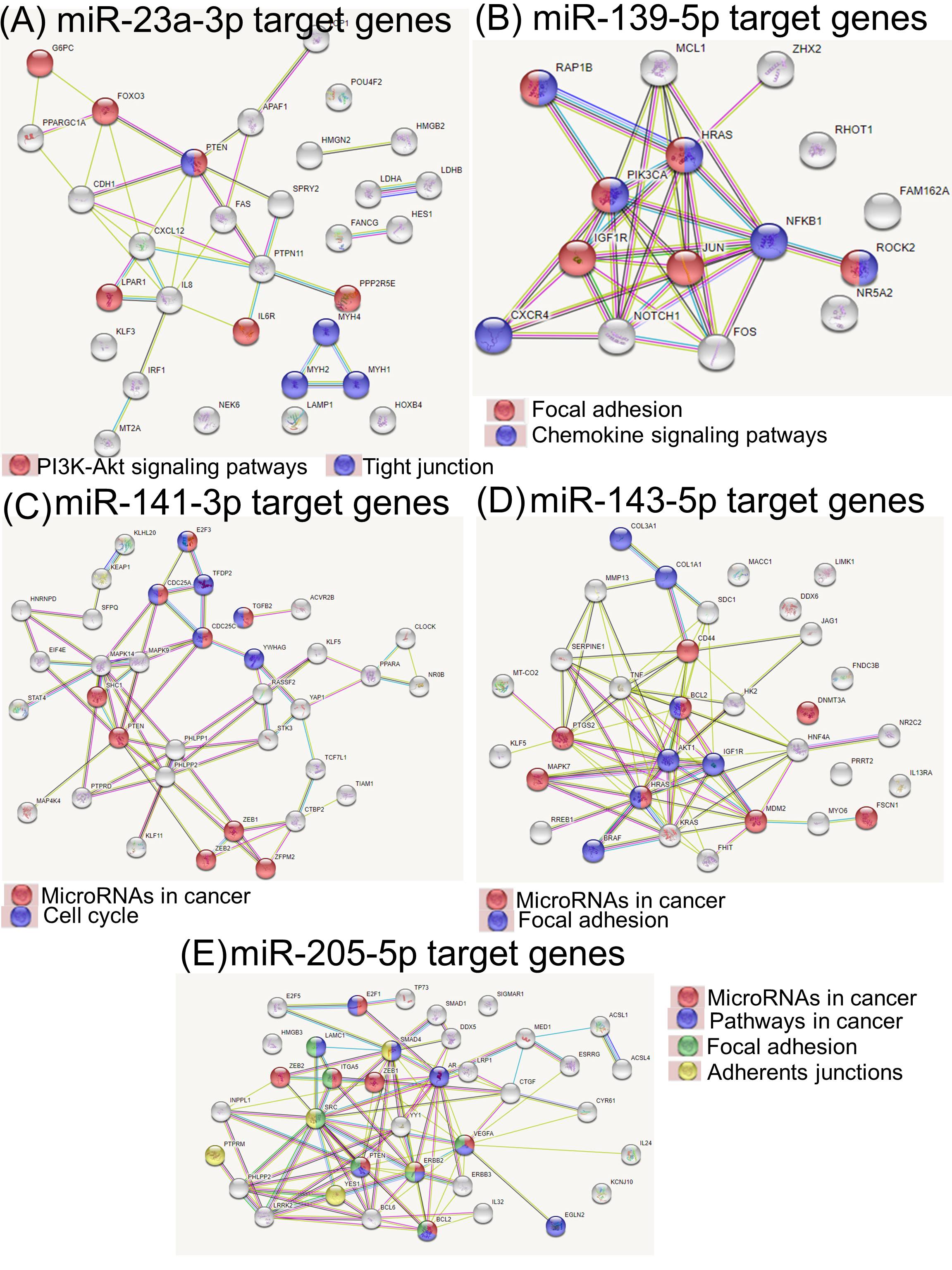

Supplement: Supplementary file 8 — Additional file 8: Figure S3. A Network analysis of the mutated genes in relation to their targeted miRNAs in bladder cancer. Schematic representation was obtained from miRnet (https://www.mirnet.ca/). [file 13046_2019_1406_MOESM8_ESM.jpg]
